# Wet Season Environments Drive Local Adaptation in the Timber Tree *Dicorynia guianensis* in French Guiana

**DOI:** 10.1111/mec.17759

**Published:** 2025-04-08

**Authors:** Julien Bonnier, Enrique Sáez Laguna, Thomas Francisco, Valérie Troispoux, Olivier Brunaux, Sylvain Schmitt, Stéphane Traissac, Niklas Tysklind, Myriam Heuertz

**Affiliations:** ^1^ BIOGECO, INRAE University of Bordeaux Cestas France; ^2^ ECOFOG, INRAE, Agroparistech, CNRS, Cirad Université des Antilles, Université de la Guyane Kourou French Guiana France; ^3^ ONF, R&D, Réserve de Montabo Cayenne Cedex French Guiana France; ^4^ UPR Forests and Societies, CIRAD Montpellier France

**Keywords:** climate change, conservation genetics, genetic diversity, landscape genetics, tropical tree

## Abstract

The vast tropical rainforests of the Guiana Shield in Northern South America play a vital role in maintaining the region's ecological balance and economy. Increasing pressure from selective logging, gold mining and climate variability threatens these ecosystems. Sustainable rainforest management requires understanding the genetic diversity and local adaptation of key tree species to inform conservation. This study focuses on *Dicorynia guianensis* (Fabaceae), a widespread and economically important tree species in French Guiana. We performed genome resequencing on 87 individuals sampled in 11 sites across French Guiana to investigate the genetic structure, diversity and genetic basis of local adaptation. Genetic structure analysis identified three distinct groups: western, central and eastern, with similar levels of genetic diversity distributed in areas with different environmental conditions. Six methods applied to detect genomic signatures of selection revealed region‐specific selective sweeps and a weak overlap between single nucleotide polymorphisms (SNPs) identified through outlier analysis or genome‐environment association analyses. The strongest associations between environmental variables and genomic constitution were observed for potential evapotranspiration of the wettest quarter and for precipitation of the coldest quarter, suggesting that environmental variables related to high rainfall during the wet season are stronger drivers of local adaptation of *D. guianensis* populations than drought. Sites located in central and western French Guiana had higher risks of climatic maladaptation. These findings advance our understanding of local adaptation and climatic vulnerability in tropical trees and emphasise the need for targeted, area‐specific management strategies for conservation and sustainable timber extraction under climate change.

## 1 Introduction

Tropical rainforests in South America, in particular the extensive biodiverse forests of the Guiana Shield, play a crucial role in regulating regional and global climates by maintaining carbon stocks and influencing hydrological cycles (Bovolo et al. 2018). French Guiana, located in the Guiana Shield between Suriname and Brazil, is a region with a remarkably continuous cover of tropical rainforests that extends across 96% of the territory (De Geyer et al. 2020). However, like the rest of the globe, French Guiana is facing the multifaceted effects of climate change, alongside anthropogenic pressures such as timber exploitation and gold mining, which are among the greatest threats to rainforest biodiversity (Delnatte and Meyer 2012; Grimaldi et al. 2015; Longueville 2022). Rapid human population growth has further exacerbated these challenges by driving urban and agricultural expansion into forested areas and increasing demand for timber resources (De Geyer et al. 2020; PRFB 2019). Current forest management in French Guiana relies primarily on natural forest exploitation under guidelines established by the Office National des Forêts (ONF), which emphasise sustainable logging practices and long harvesting cycles to maintain biodiversity and ecosystem functions (PRFB 2019). However, selective logging is focused on just five key species, including *Dicorynia guianensis* Amshoff (Fabaceae), which face disproportionate harvesting pressure. This over‐reliance risks depleting these populations and reducing genetic diversity, which could compromise forest resilience and productivity in the long term (Degen et al. 2006). Climate change is expected to exacerbate these pressures by altering precipitation patterns, lengthening dry seasons and increasing temperatures, directly influencing forest dynamics and the recovery potential of harvested forests (Bovolo et al. 2018; Guitet et al. 2014). Efforts to mitigate these impacts have included long‐term experimental research, which has provided critical insights into forest regeneration and the effects of logging on ecosystem functions (Gourlet‐Fleury et al. 2004). However, adapting forest management practices faces complex ecological and social challenges, including balancing timber production with biodiversity conservation, managing the genetic impacts of selective logging, and engaging local communities in sustainable forestry practices (Degen et al. 2006). The future of forest management in French Guiana will require adaptive strategies incorporating climate‐resilient practices, improved monitoring of logging impacts, and policy frameworks addressing both environmental and socio‐economic dimensions.

A broad body of evidence from provenance trials demonstrates (i) that most tree species are adapted to their local environments, as evidenced by adaptive differentiation of populations in quantitative traits; (ii) that local adaptation generally involves a complex polygenic architecture and (iii) that genetic variation responds to a variety of environmental drivers (de Miguel et al. 2022; Lind et al. 2018; Park and Rodgers 2023; Sáenz‐Romero et al. 2017). These experiments have mostly been conducted in boreal and temperate species, and in a few commercially important tropical trees (Aitken et al. 2008; Alberto et al. 2013). Experimental evidence of local adaptation in tropical tree species suggests that the response to environmental conditions is highly species‐specific and often compounded by fitness trade‐offs (Barton et al. 2020; Muehleisen et al. 2020; Park et al. 2010). Tropical tree species frequently have specific environmental requirements and track fine‐scale availability of water and nutrients through site topography, which explains their distribution and their phenotypic trait variation in forest landscapes (Allié et al. 2015; Engelbrecht et al. 2007; Schmitt et al. 2021). Several studies have examined fine‐scale environmental adaptation in tropical trees, confirming topography as a driver of local adaptation and observing a polygenic architecture of genotype‐environment associations (GEAs) (Brousseau et al. 2015, 2021; Schmitt et al. 2021; Tysklind et al. 2020). Regional‐scale studies of local adaptation and vulnerability to climate change in tropical trees remain scarce (but see Cruz et al. 2019; Tournebize et al. 2022) but are increasingly needed to understand the population‐specific responses and vulnerabilities of tropical trees to climate change and to design appropriate management strategies. For example, enhancing the protection of reserved areas through policy changes can significantly contribute to forest preservation, thereby promoting the ability of both natural and planted forests to adapt to climate change (Guariguata et al. 2008).

Landscape genomics provides a framework to assess local adaptation on regional geographic scales, separating adaptive effects from those of geographic distance and demographic history (Manel et al. 2010; Rellstab et al. 2015; Savolainen et al. 2013). It enables the identification of loci under selection through GEAs and allows projections of adaptive genetic variation in space to assess population risks of maladaptation. Genetic offset methods, such as Gradient Forest and redundancy analysis (RDA), estimate the mismatch between the current genomic composition of a population and the optimal composition required under future environmental conditions, highlighting populations at risk of maladaptation under climate change (Capblancq et al. 2020; Fitzpatrick and Keller 2015). These tools provide usable insights for conservation, enabling targeted management strategies. Several landscape genomics studies have succeeded in uncovering links between genomic and climate variation in plant populations (Filipe et al. 2022; Martins et al. 2018; Wang et al. 2023), or identified functional genes involved in adaptation to climate variables (Ahrens et al. 2019; Martins et al. 2018); however, such studies remain scarce in tropical trees (Barton et al. 2020; Nelson et al. 2021; Sang et al. 2022).

*Dicorynia guianensis* Amshoff (Fabaceae), locally known as Angélique, is an endemic species of the Guiana Shield. It is one of the 16 hyperdominant species in the Amazon basin in terms of wood productivity and biomass (Fauset et al. 2015) and preferentially occurs in low elevations (< 200 m) on clayey or sandy soils of ‘terra firme’ rainforests, less commonly in seasonally inundated areas (Falcão et al. 2022). In French Guiana, *D. guianensis* accounts for 54% of timber production and is mainly used for carpentry, joinery, flooring and harbour construction (Flora 2018; Guitet et al. 2014). This selective logging is putting significant pressure on the species, which is of particular concern given its sensitivity to drought. Studies have shown that drought reduces its growth rate (Baraloto et al. 2006), impairs water transport due to low resistance to stem, branch and leaf xylem embolism (Fortunel et al. 2023; Ziegler et al. 2019), and limits its ability to maintain leaf turgor pressure (Maréchaux et al. 2015). The reproductive ecology of *D. guianensis* further highlights its vulnerability. Pollination is primarily facilitated by large bees, with pollen dispersing an average of 200 m from the source tree, while seed dispersal is limited to short distances by gravity and wind, rarely exceeding 50 m (Caron et al. 1998; Forget 1988; Jésel 2005). Its populations are often distributed in genetically differentiated aggregates, typically occupying less than one hectare, with mature tree densities ranging from 4 to 14 per hectare (Bariteau 1993; Gourlet‐Fleury et al. 2004). Population genetic structure has been observed across French Guiana, with a significant differentiation between eastern and western geographical groups, potentially influenced by environmental gradients such as precipitation differences (Bonnier et al. 2024; Caron et al. 2000).

Using genome resequencing in 87 individuals from 11 sites widespread across coastal French Guiana, we conducted a population and landscape genomics study of *Dicorynia guianensis* to characterise its genomic variation across environmental gradients, assess local adaptation and its drivers and evaluate its genomic vulnerability to climate change. We applied the IPCC (2007) vulnerability framework to evaluate the vulnerability of *Dicorynia guianensis* populations to climate change. This framework conceptualises vulnerability as a function of three components: exposure, sensitivity and adaptive capacity (Locatelli et al. 2008). We considered exposure and sensitivity together by calculating the genomic offset (Fitzpatrick and Keller 2015). Adaptive capacity was proxied by the genetic diversity within populations, which influences their ability to adapt to environmental changes. We addressed the following questions:

- Does the genomic dataset reveal new insights into the population genetic structure and into the demographic history of *Dicorynia guianensis* in French Guiana?
- Can we detect genomic signatures of selection between populations of *Dicorynia guianensis* in French Guiana?
- Can we detect loci associated with environmental variation, suggesting local adaptation? Which environmental variables are the strongest drivers of these putative adaptations?
- How vulnerable are *D. guianensis* populations to climate change? Specifically, do they show differences in (1) adaptive capacity using genetic diversity as a proxy, (2) sensitivity using inbreeding and (3) risk of maladaptation using a genomic offset approach?

## 2 Materials and Methods

### 2.1 Sampling and Study Sites

**FIGURE 1:**
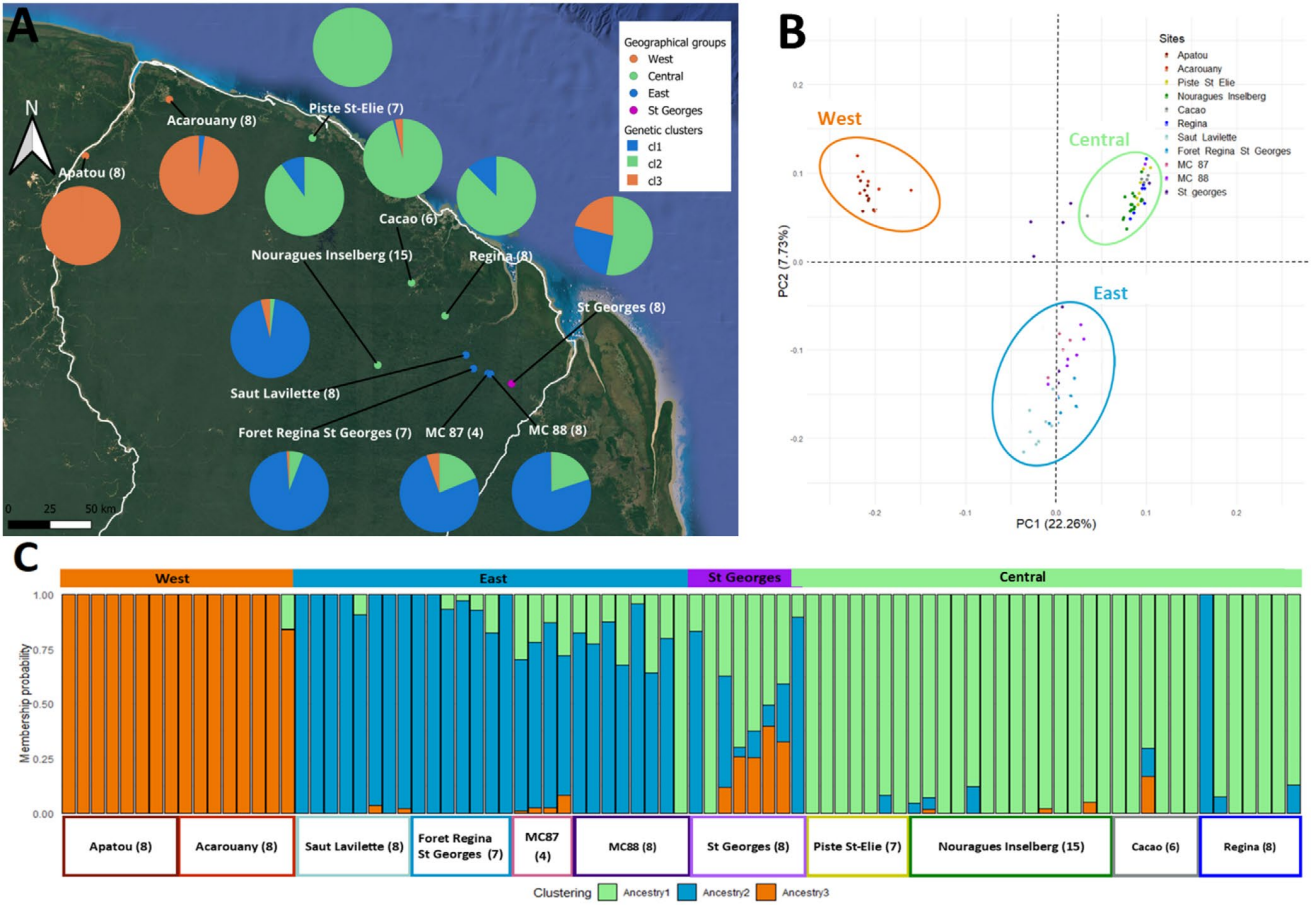
(A) Geographical genetic structure of the 11 sampling sites of *Dicorynia guianensis* in French Guiana assembled in four geographical groups with the number of individuals (*N*) in each site. (B) Principal component analysis (PCA) conducted on the sampling sites using the first two components (PC1 and PC2). The ellipses showing the groups have been added subsequently to make the graph easier to read. (C) ADMIXTURE estimates of population structure in *Dicorynia guianensis*. Each vertical line represents one individual, and colour segments represent its estimation of individual ancestry proportions in each of *K* = 3 genetic clusters.

The *D. guianensis* tissue samples were collected during several past projects of the UMR EcoFog laboratory (Treemutation, COUAC and Dygepop), and stored in silica gel at air‐conditioned room temperature until DNA extraction. Leaf and cambium samples were collected from 11 sampling sites, most of which were located within the Guianese coastal forest: St‐George (7), Saut Lavilette (8), Regina (8), Forêt de Régina St Georges (8), MC 87 (4), MC 88 (8), Cacao (6), Nouragues Inselberg (15), Piste St‐Elie (7), Acarouany (8) and Apatou (8), representing a total of 87 individual samples (Figure 1A). All sampled sites are part of the Domaine Forestier Permanent (DFP) managed by the ONF for conservation and sustainable timber production using reduced impact logging (Guitet et al. 2014). Through the sampling range, annual rainfall varies between 2000 mm in the northwest at Acarouany and Apatou, and 3600 mm in the southwest at Regina and Forêt de Regina St Georges sampling sites (Figure S1).

### 2.2 Environmental Variables

The environmental variables used here were extracted from three databases: BIOCLIM, ENVIREM and Guyane SIG. WorldClim is an interpolated climate data set of high spatial resolution (about 1 km^2^), comprising 19 variables mainly related to precipitation and temperature collected over a target time range of 1970–2000 (Fick and Hijmans 2017). ENVIREM is a set of 16 climate variables and two topographical variables of similar resolution to WorldClim, many of which are likely to have a direct bearing on the ecological or physiological processes that determine species distributions (Title and Bemmels 2018). Guyane SIG (www.guyane‐sig.fr) is a regional GIS platform for land analysis, planning, and development, providing maps of the territory's composition. For our study, we obtained maps concerning the soil components of French Guiana: pedology, lithography, soil formations and forest habitat types (Guitet, Olivier, et al. 2015).

Variables for future climate conditions used to assess the risk of climate maladaptation (section 2.9) were either extracted from Worldclim or calculated following Envirem recommendations and followed by data standardisation (Title and Bemmels 2018). As future data, we used the mean values of four global climatic models (GCM): IPSL‐CM6A‐LR (Boucher et al. 2020), MPI‐ESM1‐2‐HR (Gutjahr et al. 2019), MRI‐ESM2‐0 (Yukimoto et al. 2019) and UKESM1‐0‐LL (Sellar et al. 2019), under two distinct climate forcing socio‐economic pathways (SSP) scenarios: a moderate emissions scenario (SSP2‐4.5) and a more severe scenario (SSP3‐7.0; Lee et al. 2023). The projections were calculated for the 2041–2060 time interval at a high spatial resolution of approximately 1 km^2^ (30 arc‐seconds).

### 2.3 Laboratory Procedures

For each leaf and cambium sample, genomic DNA was extracted from 50 mg of silica‐dried material. Each sample was independently ground into a fine powder with a Genogrinder 2010 (Spex SamplePrep, NJ, USA) and a CTAB‐based DNA extraction protocol was employed following Doyle and Doyle (1990). The DNA concentration of the samples was assessed by absorbance using a NanoDrop 8000 (Thermo Fisher Scientific, MA, USA) spectrophotometer. Extractions with a concentration lower than 30 ng/μL were repeated until reaching a satisfactory concentration. Genomic libraries (*n* = 92, including a few repetitions) were constructed at the GENOSCOPE (Evry, France) facility, following a customised protocol based on the NEB DNA Library Prep Kit for Illumina. An essential purification step was performed prior to DNA fragmentation to remove small fragments identified during initial quality testing, which resulted in a loss of approximately 50% of DNA concentration, but was essential to produce high‐quality libraries. Sequencing was performed using Illumina Flow cell S4 2 × 150 bp V1.5 PE sequencing on a NovaSeq 6000 Sequencing System, aiming for a 10× coverage based on a genome size of 550 Mbp (Schmitt et al. 2024).

### 2.4 Genomic Data Processing

Quality filtering of sequencing data was performed with fastq v.0.23.2 (Chen 2023) by trimming sequencing adapters and removing reads shorter than 100 bp (duplicate accuracy calculations = 2; removal of poly‐G; minimum read length = 100). BWA‐MEM (Li 2011; parameters: ‐ mem ‐M) was used to align reads to the *D. guianensis* reference genome (Schmitt et al. 2024). GATK v4.2.6.1 (DePristo et al. 2011) with HaplotypeCaller and GenotypeGVCFs tools was used to call single nucleotide polymorphisms (SNPs) following the GATK best practices workflow for variant discovery (Germline Short Variant Discovery, 2024). BCFtools v1.9 (Li 2011) was used to select bi‐allelic SNPs which were quality filtered using GATK VariantFiltration and SelectVariants tools (QUAL < 50.0, QD < 2.0, FS > 60.0, SOR > 3.0). PLINK v2.00a4 (Purcell et al. 2007) was then used to exclude all SNPs with missing values (geno = 0; mind = 0.1). To assess the most relevant genetic variation in this extensive dataset, we located these SNPs with respect to the transposable elements of the reference genome using bedtools v2.30.0 (Quinlan and Hall 2010) with the *intersect* command and PLINK v2.00a4 with *extract*, keeping only those SNPs that were located outside of transposable elements. The filtered dataset of 11,466,201 SNPs in 87 individuals was used for the detection of selective sweeps and for demographic history analysis (see below). These analyses benefit from using the full linkage structure of the dataset, for example, maximising sensitivity to detect regions under selection (Vitti et al. 2013). For analyses sensitive to linkage disequilibrium (LD), including genetic structure, outlier detection, GEAs and genomic offset analyses, LD pruning was conducted using *PLINK* v2.00a4 (*r* ^2^ = 0.4; window size = 10,000 bp; step size = 5000 bp), resulting in a dataset of 599,959 high‐quality SNPs genotyped in 87 individuals. LD pruning reduces redundancy among SNPs, prevents inflation of associations and ensures accurate representation of genetic variation in population‐level analyses (Purcell et al. 2007). Genetic relationships between individuals were assessed using the KING tool in PLINK v2.00a4, which calculates kinship coefficients between all pairs of individuals in order to verify the absence of close family links that could bias the population structure analysis.

### 2.5 Population Genetic Structure, Diversity and Demographic History

We assessed the genetic structure of populations using ADMIXTURE v1.3.0 (Alexander et al. 2009), a program that models the probability of observed genotypes using ancestry proportions in *K* genetic clusters (i.e., populations) based on a maximum likelihood approach. For each *K* ranging from 1 to 10, we ran the software with default settings. The most probable value of *K* was determined using the cross‐validation (cv = 100) method implemented in ADMIXTURE (Figure S2). In addition, we also performed principal component analysis (PCA) to infer the number of genetic clusters with PLINK v2.00a4 (Purcell et al. 2007) and R package ggplot2 (Wickham 2009) for visualisation of the results. *F*_ST_ values among the 11 sampling sites were calculated using the R package *Hierfstat* (Goudet 2005). Based on the genetic structure observed (see Results below), genetic diversity analyses were then carried out on three groups (West, Central and East), and separately on St Georges. With *Hierfstat*, values for expected heterozygosity (*H*_E_), observed heterozygosity (*H*_O_), inbreeding coefficient (*F*_IS_), and allelic richness (*A*) were calculated for each group. Nucleotide diversity (*π*) was computed using the R package *pegas* (Paradis 2010). Deviation from Hardy–Weinberg equilibrium (HWE) was tested using the *hw.test* function, and *p* were obtained for loci and populations with 1000 permutations. To assess the demographic history, Tajima's *D* and its *p* were calculated using the tajima.test function in *pegas*, and SMC++ (Terhorst et al. 2017) was used to produce stairway plots of effective population size *N*_E_ through time for three groups (West, Central, and East), based on the 20 longest super‐scaffolds. Calculation of Tajima's *D* was not applicable to the St Georges site as the sample size was too small.

### 2.6 Genomic Signatures of Selection

Detecting loci under selection is a complex task, and for robust detection, it is advised to use multiple methods as no single method consistently performs better in all scenarios due to differing assumptions (Feng and Du 2022). To identify loci potentially under selection, we used three approaches: detecting selective sweeps, identifying outlier loci based on genetic differentiation among sites (*F*_ST_), and identifying outlier loci based on their position along principal components of population structure. Selective sweeps across the *D. guianensis* genome were identified using SweeD software (v4.0.0; Pavlidis et al. 2013), which uses the composite likelihood ratio (CLR) model to analyse SNP site frequency spectra, which were obtained from the dataset before LD pruning. SweeD analyses were performed on the three genetic groups (see Results) to observe variations in the occurrence of selective sweeps along the genome among these groups. CLRs were averaged within a 10 kb window, setting the ‐grid parameter according to scaffold lengths, across 20 largest super‐scaffolds. The following outlier analyses were carried out considering the 11 sampling sites, each subject to different environmental conditions. *F*_ST_ outlier detection was conducted using BayeScan version 2.1 (Foll and Gaggiotti 2008). BayeScan uses a Markov chain Monte Carlo algorithm to detect *F*_ST_ outlier loci against background values of population differentiation (*F*_ST_) among sampling locations. Bayescan was run for a total of 50,000 iterations with a burn‐in of 5000, preceded by 20 pilot runs of 100 iterations, and the prior odds for the neutral model were 100. The R package *pcadapt v.4.3.5* (Luu et al. 2017) was also used to detect genetic markers potentially involved in the local adaptation of *D. guianensis* . The optimal number of principal components (*K*) was determined by performing a PCA. Test statistics were calculated for each SNP using the *z*‐scores obtained when regressing SNPs on the first three retained principal components. To identify outlier SNPs from *pcadapt* and BayeScan results, *p* were transformed into *q*‐values using the R package *qvalue v.2.36.0* (Storey et al. 2024/2014) to control for false positives (FDR). Markers with *q*‐values below 0.05 were considered outliers.

### 2.7 Genotype‐Environment Association Analysis

To identify the most relevant environmental variables for GEA in *D. guianensis* while avoiding model overfitting, we employed a predictive variable reduction approach using RDA with forward selection (Capblancq and Forester 2021). Starting with a null model where genetic variation is explained by a fixed intercept, variables were added sequentially using the ordiR2step function in the vegan package (Dixon 2003). The inclusion of each variable was based on its statistical significance (*p* < 0.01) determined through 999 permutations. To further limit correlation in the explanatory variables, a PCA was carried out on 31,330 points systematically sampled in French Guiana for each of the variables retained by RDA with forward selection. For pairs of variables that were highly correlated in the PCA, a single variable was retained using a correlation threshold of |*r*| > 0.7, so that the variables retained were as little collinear as possible. This threshold was chosen as it is a commonly accepted standard in multivariate analyses to address multicollinearity and was consistently applied across all sets of correlated variables to maintain uniformity (Legendre and Legendre 2012; Zuur et al. 2010). The procedure resulted in five environmental variables retained for GEA (see Results). To identify loci putatively under environmental selection, we conducted GEA analyses using BayPass version 2.2, a robust framework for detecting adaptive SNP signals (Gautier 2015). To perform BayPass analyses, a population covariance matrix (*Ω*) was constructed to account for the confounding effects of population structure, and Bayes factors (BFs) were calculated to measure the strength of correlation between each SNP and each environmental covariate. BFs values were calculated on all SNPs for the selected environmental variables using the standard covariate model (STD). SNPs with BFs above 15 dB (decibel units) were considered outliers. A second GEA analysis was carried out using a latent factor approach, latent factor mixed modelling (LFMM), with the R package *lfmm2* (Caye et al. 2019). Three confounders were taken into account to control for population structure (*K* = 3), selected based on the ADMIXTURE analysis described in 2.6. We used a False Discovery Rate (FDR) of 5% by applying the Benjamini‐Hochberg algorithm (Benjamini and Hochberg 1995) to select associated SNPs, using the R package *qvalue v.2.36.0* (Storey et al. 2024/2014). RDA was performed using the R package *vegan* v.2.6.4 (Dixon 2003), treating the selected SNPs as response variables and environmental variables as explanatory variables. We adopted the method in which outlier loci are identified on the basis of their extremity along a Mahalanobis distance distribution using the two first axes (*K* = 2) of RDA (Capblancq et al. 2018; Capblancq and Forester 2021). The *p* were adjusted to an overall significance level of 0.05 using Bonferroni's method. At the completion of these analyses, a Venn diagram using the R package *ggVennDiagram* (Gao et al. 2021) was produced to visualise the overlap between the SNPs detected as differentiation outliers and those putatively under environmental selection identified by the different methods used. In addition, to disentangle the environmental drivers of genetic variation among sample sites and assess their relative importance, partial RDAs were performed considering environmental variables (five variables retained, see Results), geography and population genetic group membership.

### 2.8 Genomic Contexts of Candidate SNPs

To understand the functions of the genes associated with the SNPs identified as outliers, we carried out a Gene Ontology (GO) analysis using the python programs *pandas* and *gffutils* (Dale n.d.; Mckinney 2011). We used the reference genome of *D. guianensis* , with functional annotations by Schmitt et al. (2024). SNPs conserved by at least four outlier selection methods were extracted and associated with their nearby genes (max distance = 1000 bp). The associated genes were filtered to obtain the corresponding GO annotations and quantify the different types of biological functions, cellular processes and cellular components associated with the outlier genes. A gene enrichment analysis was also performed with the R package topGo (Alexa and Rahnenfuhrer 2016; Subramanian et al. 2005) to determine which GO terms are over‐represented, indicating an association with the selected genes. The significance of the results was obtained using Fisher's tests (*p* < 0.01).

### 2.9 Vulnerability to Climate Change

The risk of maladaptation to climate change was investigated using the concept of genomic offset, introduced by Fitzpatrick and Keller (2015). Higher values indicate a greater mismatch between current and predicted future genomic compositions, suggesting a higher potential maladaptation to changing climates. Genomic offset predictions were calculated in several steps. First, we computed an ‘adaptively enriched’ RDA, identifying associations between loci previously detected as putatively under selection and climatic variables resulting from the reduction of variable space (see 2.8), using RDA following the methodology of Capblancq and Forester (2021). TWI was not used for these calculations because it is not possible to have future estimates of this environmental variable, which is not affected by climate variation. This relationship was built using the allelic frequencies for the set of 459 SNPs putatively under selection retained by at least four methods (see Results) and the current climatic conditions across the 11 sampled localities. In a second step, we projected this relationship through space and time across each pixel of the DFP to estimate the current and future genomic compositions. To ensure accurate comparison between current and future genomic compositions, all present and future climatic variables used in the projections were standardised (i.e., subtracting the mean and dividing by the standard deviation). We also verified that the climatic conditions of the sampled localities covered the main part of the climatic range of the DFP to guarantee that the model was not projected onto extensively different climatic conditions where it had not been trained. Finally, we calculated the Euclidean distance between the two genomic compositions, later referred to as genomic offset. The genomic offset predictions were made for the 2041–2060 time frame under SSP2‐4.5 and SSP3‐7.0. The relatively close time frame to the present day was selected to address the genomic offset framework assumption that GEAs derived from past or current genomic and climatic data will remain valid over time (Rellstab et al. 2021). Indeed, we assumed that, due to the relatively long generation time of *Dicorynia guianensis* (PRFB 2019), demographic turnover would only be partial and changes in the environment due to local socio‐economic development in French Guiana (not captured by the models) remain moderate, limiting the advent of potentially new advantageous mutations, migration, or admixture.

To assess the adaptive capacity, the diversity statistics (*H*_E_, *H*_O_, *F*_IS_, and *A*_R_) were calculated with the R package *Hierfstat* (Goudet 2005) and nucleotide diversity (*π*) with *pegas* (Paradis 2010) on the SNPs putatively under selection retained by at least four methods for each sampling site and each group.

## 3 Results

### 3.1 Population Genetic Structure, Diversity and Demographic History

The kinship coefficients calculated from the KING analysis showed that pairs of individuals had values close to zero, suggesting a weak genetic relationship among individuals, and thus confirming the absence of close family ties in the sample. Genetic structure analysis with ADMIXTURE resulted in *K* = 3 genetic clusters as the clustering solution with the lowest cross‐validation error. The three clusters corresponded to sampling sites located in the west (Acarouany and Apatou), in the central region (Piste St‐Elie, Nourague Inselberg, Cacao, and Regina) and in the east (Saut Lavillette, Foret Regina St Georges, MC 87, and MC 88) of French Guiana, with individuals from the easternmost site of St Georges appearing as admixed (Figure 1C). The PCA supported ADMIXTURE results, identifying a pronounced differentiation between sites in the west of French Guiana (Acarouany and Apatou) and all others, emphasised by the PC1 score of 22.26%. Central and eastern sites were differentiated along PC2, with PC2 explaining 7.73% of the dataset's variation. Individuals from St Georges displayed a large variation in the PCA plot, overlapping with the group formed by eastern sites and spreading towards the one formed by central sites (Figure 1B). The revealed genetic structure led us to merge sampling sites into three genetic groups based on geographic and genetic proximity (Figure 1C). St. Georges was excluded from these groups due to its highly complex and distinct genetic structure, which did not align with the patterns observed at the other sites. This complexity likely reflects unique historical or ecological factors affecting St. Georges, which cannot be addressed here due to low sample size but warrants further investigation. The strong differentiation between western and other sampling sites was confirmed based on differentiation analysis, with a mean average *F*_ST_ of 0.110 for Acarouany and 0.116 for Apatou versus all other sites (Table S2).

Genetic diversity analyses revealed similar values for *D. guianensis* groups for allelic richness (*A*_R_), ranging from 1.60 (Central) to 1.65 (West), *H*_O_ ranging from 0.179 (Central) to 0.199 (West) and *H*_E_ ranging from 0.203 (Central) to 0.223 (West) (Table 1). Moderate inbreeding and negative Tajima's *D* values were identified for all groups (Table 1). Stairway plots (Figure S3) from the three groups showed a bottleneck ca. 100,000 years ago, from which central and eastern, but not western sites recovered about 30,000 years ago. Effective population sizes were stable since recovery and largest in the East, followed by the central region and the West.

**TABLE 1:** Genetic diversity and population demographic history statistics for three observed geographical groups (+ St Georges site) of *Dicorynia guianensis*.

Geographical groups	*N*	*A*_R_	*H*_O_	*H*_E_	*F*_IS_	Tajima's *D*	*π*
West	16	1.65	0.199	0.223	0.097*	−1.90*	0.009
Central	36	1.60	0.179	0.203	0.113*	−1.86*	0.004
East	27	1.63	0.180	0.213	0.140**	−1.92*	0.005
St Georges	8	1.64	0.187	0.219	0.115	−2.36	0.014
Overall	87	1.63	0.186	0.215	0.116*	−1.31	0.016

Abbreviations: *A*_R_, allelic richness standardised to a sample size of 12 gene copies; *F*_IS_, inbreeding coefficient; *H*_E_, expected heterozygosity; *H*_O_, observed heterozygosity; *N*, sample size; *π*, nucleotide diversity per nucleotidic site.

* *p* < 0.05.

** *p* < 0.01.

### 3.2 Identification of Loci Putatively Under Selection and Their Environmental Drivers

The search for selective sweeps identified 348 SNPs in the western group, 385 SNPs in the central group, and 416 SNPs in the eastern group, with CLR values greater than zero. All SNPs with CLR > 0 were represented by Manhattan plots (Figure S4), showing variability in sweep positions at the highest CLR values between groups. 318 SNPs were identified as only present in the western group, 367 in the central group, and 376 unique to the eastern group. The western and eastern groups had the highest number of SNPs affected by common selective sweeps (*n* = 25; Figure S5).

A total of 22,819 SNPs were identified by the five methods applied for the detection of loci under selection (BayeScan, pcadapt, LFMM2, Baypass and RDA), representing 3.8% of the initial dataset of 599,959 SNPs retained after filtering and linkage pruning steps. Outlier detection methods based on genetic differentiation only (pcadapt and BayeScan; see Figure S6) detected 1004 SNPs in common between the two methods, and methods based on GEAs (LFMM2, Baypass and RDA) detected 229 SNPs in common among methods. GEA analyses were conducted based on the following five environmental variables resulting from the variable reduction procedure: aridity index (AI), mean temperature of the coldest quarter (BIO11 and MTCQ), topographic wetness index (TWI), average monthly potential evapotranspiration (PET) of the wettest quarter (PETWQ) and precipitation of the coldest quarter (BIO19, PCQ). Soil composition variables did not show a significant association with the SNPs considered, probably due to the great heterogeneity of soil and subsoil types in the sampling sites, which may have masked consistent patterns. For BayPass, the SNP numbers reported correspond to the sums of SNPs identified by the five single‐variable GEAs. Fifty‐one SNPs were identified as overlapping among all five methods. Details of numbers of SNPs identified as under selection by a single method or by the overlap of different combinations of methods can be observed in Figure S7A. Pcadapt identified the highest number of outlier SNPs, including 8366 unique outliers that were not identified by any other methods; BayPass identified the lowest number of SNPs as under selection. The SNPs detected by at least four methods (459 SNPs) were retained for GO analysis.

**FIGURE 2:**
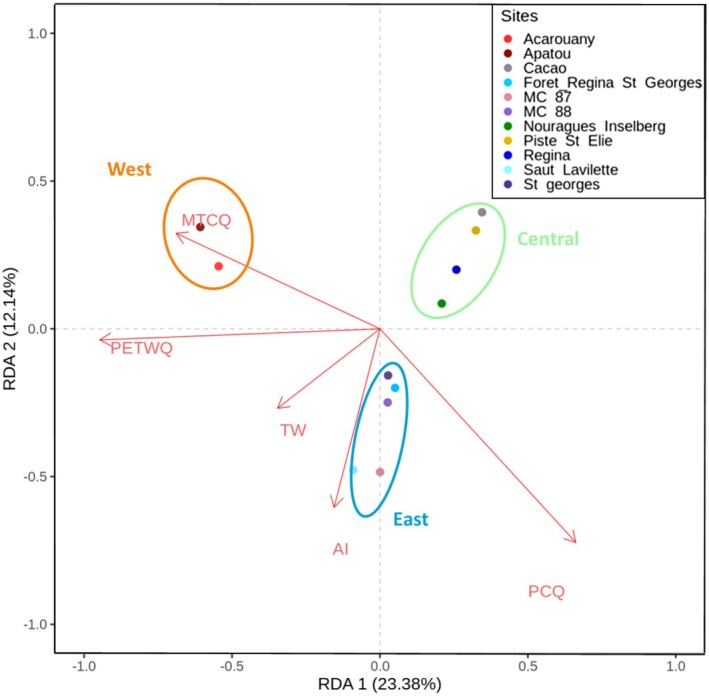
Biplot of redundancy analysis (RDA) illustrating the strength of association between environmental variables and genetic variation at 11 study sites for *Dicorynia guianensis* in French Guiana. RDA is a multivariate analysis that plots SNPs, individuals and/or sampling sites, as well as explanatory variables, along canonical axes. Variable names: AI, Aridity Index; MTCQ, Mean Temperature of the Coldest Quarter; PCQ, Precipitation of the Coldest Quarter; PETWQ, Potential Evapotranspiration of the Wettest Quarter; TWI, Topographic Wetness Index.

**FIGURE 3:**
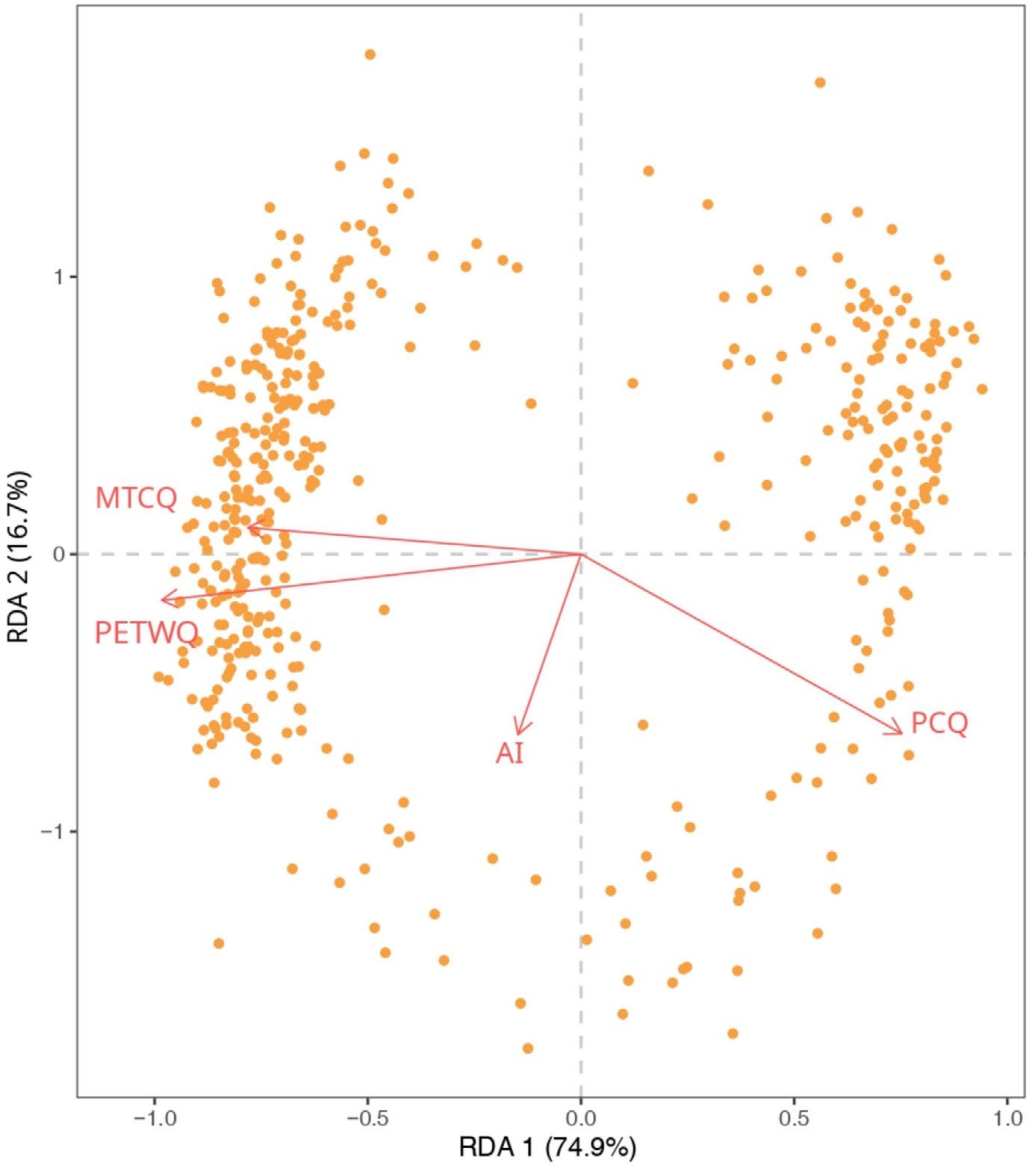
Association between putatively adaptive loci of *Dicorynia guianensis* (459 SNPs detected by at least four methods) and climatic variables identified as drivers of adaptation used to compute genomic offset. The figure highlights the relationship between loci identified as climate‐associated and specific environmental variables, emphasising the climatic variables most strongly associated with genetic variation. Variables named: AI, Aridity Index; MTCQ, Mean Temperature of the Coldest Quarter; PCQ, Precipitation of the Coldest Quarter; PETWQ, Potential Evapotranspiration of the Wettest Quarter.

The partial RDA (pRDA) (Table 2) showed that the complete model, integrating environment, geography, and genetic group membership, explained 18% of the total genetic variance in the dataset. The model assessing the pure effect of the environment (i.e., controlling for geographic location and genetic structure) explained 6% of the total genetic variance, while the model assessing the pure effect of geographic location explained 2%. The RDA biplot shows the correlation between genetic variation among sampling sites and environmental predictors (Figure 2). The first axis of the biplot, which accounted for 23.4% of variation, was mainly associated with potential evapotranspiration in the wettest quarter (PETWQ) and mean temperature in the coldest quarter (MTCQ), while the second axis (12.1%) was mostly determined by the AI. Precipitation of the coldest quarter (PCQ) and topographic wetness index (TWI) equally contributed to both axes. The western sampling sites, Apatou and Acarouany, were the most impacted by the RDA1 axis: they experienced the lowest precipitation and the highest potential evapotranspiration during the coldest/wettest quarter, that is, the rainy season (Table S1). The separation between eastern and central sites appeared more determined by the RDA2 axis and the AI, with less humid conditions in the central (lower AI, lower PCQ) than in the Eastern group (Figure 2). BayPass results confirmed the role of rainy season variables as selective drivers, with the highest number of loci associated with PETWQ (1031 SNPs) and the second‐highest with PCQ (744 SNPs; Figure S7B). The 459 SNPs detected as putatively under selection by at least four methods were plotted against the four environmental variables identified as the strongest putative drivers of adaptation (Figure 3). This revealed a greater proportion of SNPs co‐varying on axis 1 associated with PETWQ and MTCQ and a smaller proportion on axis 2 associated with the AI.

**TABLE 2:** The influence of environment, geography and genetic cluster membership on genetic variation among sampling sites in *Dicorynia guianensis*, decomposed with pRDA (partial redundancy analysis) after 999 permutations. Inertia is analogous to variance.

Partial RDA models	Inertia	*R* ^2^	*p* (> *F*)	Proportion of explainable variance	Proportion of total variance
Full model: *F* ~ env. + geog. + genet.	1204.6	0.071	0.001	1.00	0.18
Pure environment: *F* ~ env.|(geog. + genet.)	418.2	0.008	0.001	0.35	0.06
Pure genetic distance: *F* ~ genet.|(env. + geog.)	350	0.021	0.001	0.29	0.05
Pure geography: *F* ~ geog.|(env. + genet.)	161.3	0.003	0.003	0.13	0.02
Confounded environment/genetic distance/geography	929.5			0.77	0.14
Total unexplained	5539.8				0.82
Total inertia	6744.4				1.00

### 3.3 Gene Ontology

In total, 126 SNPs were mapped to single known genes, and most of them were assigned to the ‘biological process’ category in the GO analysis, which represented 56% of GO terms identified (2975 GO terms). The results of the GO enrichment analysis show greater significance and larger numbers of GO terms relating to biological processes (Figure S8). This emphasises responses to organic substances, oxygenated compounds and endogenous stimuli, suggesting diversified biological activity and response to various environmental stimuli in genes containing outliers.

### 3.4 Vulnerability to Climate Change

**FIGURE 4:**
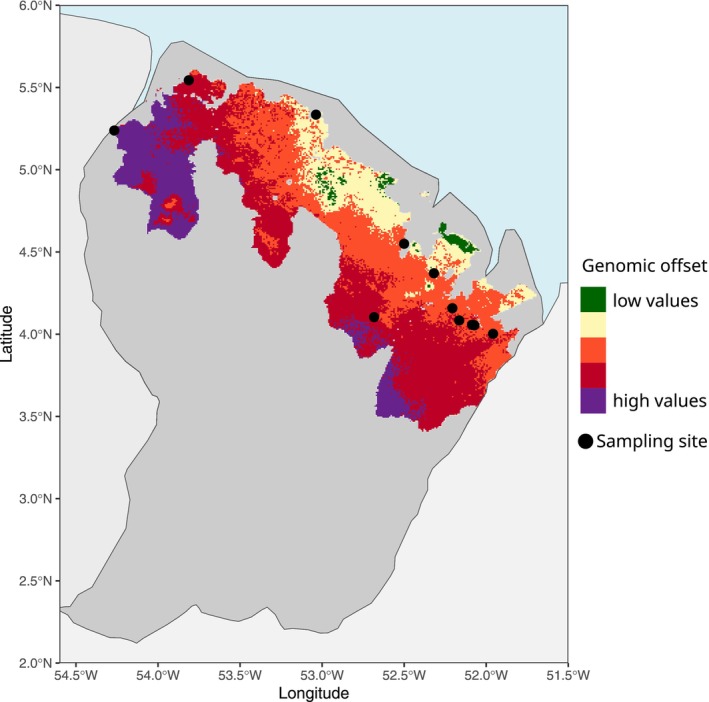
Spatial projection of the genomic offset in *D. guianensis* across the Permanent Forest Domain (DFP) in French Guiana for the 2041–2060 time frame under the Shared Socio‐economic Pathway (SSP) 2–4.5, a moderate emissions scenario.

The climatic conditions of the sampled localities were spread within the climatic range of the DFP, validating the area of application of the model (Figure S9). We also computed genomic offset for a more restricted area where climate conditions of sampled sites had enhanced representativity. The obtained genomic offset values were very similar to the ones obtained for the DFP. The RDA assessing the association of the genetic constitution of sampling sites for putatively adaptive loci of *D. guianensis* and the four key environmental variables (AI, MTCQ, PETWQ and PCQ identified with GEA analysis) produced a representation similar to that obtained for all the SNPs studied by RDA (compare Figure S10 with Figure 2). The risk of maladaptation, computed as genomic offset, showed the highest values for the western site of Apatou and the central site of Nouragues Inselberg (Figure 4). A gradient of increasing risk of maladaptation was identified from the coast of French Guiana towards the interior and the west of the DFP for the two different models SSP2‐4.5 and SSP3‐7.0 (Figures 4 and S11), driven predominantly by shifts in precipitation (PCQ) and PETWQ (Figure S10). These variations are influenced by projections assuming an increase in extreme rainfall during the wet season and extreme high temperatures during the dry season in South America.

Estimation of adaptive capacity based on genetic diversity statistics on loci potentially under selection showed lower diversity for the west (*A*_R_ = 1.62, *H*_E_ = 0.19, *π* = 0.073) than for the central (*A*_R_ = 1.89, *H*_E_ = 0.33, *π* = 0.128) or in the east (*A*_R_ = 1.92, *H*_E_ = 0.38, *π* = 0.126, Table S3). The western site of Apatou that had the highest genomic offset had also the lowest adaptive capacity based on loci putatively under selection.

## 4 Discussion

We identified moderate population genetic structure, distinguishing western, central, and eastern geographical groups of *D. guianensis* in French Guiana, and a complex population genetic structure in the easternmost site of St. Georges. The environmental variables identified as the strongest drivers of genetic variation in our dataset, for example, those with the highest numbers of co‐varying SNPs (Baypass analysis), were related to excess moisture during the rainy season, namely potential evapotranspiration during the wettest quarter, PETWQ, and precipitation of the coldest quarter, PCQ. Mean temperature during the coldest quarter (MTCQ) showed some co‐variation with PETWQ in the RDA biplot but had fewer co‐varying SNPs. The AI and the topographic wetness index completed the set of the five most important environmental explanatory variables for genetic variation in this drought‐sensitive species. Although all sites displayed similar levels of genetic variation, they were not equal with respect to climatic maladaptation and adaptive capacity: the most vulnerable sites with respect to future climate were located away from the coast, and especially in the west of the Domaine Forestier Permanent.

### 4.1 Genetic Structure, Diversity and Demographic History

*Dicorynia guianensis* featured moderate genetic diversity in genetic groups in French Guiana (*H*_O_ = 0.179–0.199; *H*_E_ = 0.203–0.223), when compared with SNP analyses in other tropical tree species in French Guiana such as *Jacaranda copaia* (*H*_O_ = 0.095; *H*_E_ = 0.192), *Dipteryx odorata* (*H*_O_ = 0.330; *H*_E_ = 0.250) or *Carapa guianensis* (*H*_O_ = 0.262; *H*_E_ = 0.251) (Capo et al. 2024; Honorio Coronado et al. 2020; Tysklind et al. 2019). The similarity of allelic richness (*A*_R_) and heterozygosity (*H*_O_ and *H*_E_) values between genetic groups indicates a relatively homogeneous distribution of genetic diversity. Nucleotide diversity (*π*) values for *D. guianensis* ranged from 0.004 to 0.014 across geographical groups, with the lowest diversity observed in the Central group and the highest in St. Georges. Comparatively, a study on European forest tree species reported nucleotide diversity values ranging from 0.003 to 0.007 (Milesi et al. 2024). This suggests that *D. guianensis* exhibits nucleotide diversity levels comparable to or slightly higher than those observed in these European species. Significant inbreeding coefficient (*F*_IS_) values for the West, Central and East groups suggest some inbreeding, potentially resulting from the merging of different local samples resulting in non‐panmictic groups in the analyses (Wahlund effect).

The genetic structure revealed by our whole genome resequencing dataset confirms and deepens the results of two previous studies on *D. guianensis* populations across French Guiana using chloroplast haplotypes and nuclear microsatellites (Bonnier et al. 2024; Caron et al. 2000). The marked differentiation between western and central or eastern groups in French Guiana (*F*_ST_ = 0.147) observed in our study was paralleled in *Jacaranda copaia* (*F*_ST_ = 0.120), *Hymenaea courbaril* (*F*_ST_ = 0.213) and *Vouacapoua americana* (*F*_ST_ = 0.195; Capo et al. 2024; Chaves et al. 2018; Dutech et al. 2004), but not in *Eperua falcata*, where differentiation was less pronounced at the regional level, *F*_ST_ = 0.032 (Brousseau et al. 2021). Our study disclosed two genetically differentiated groups in the centre and east of French Guiana in *D. guianensis* , not revealed in previous studies (Bonnier et al. 2024). This pattern of genetic differentiation reflects historical processes shaped by climatic fluctuations and geographic isolation (Barthe et al. 2017; Dutech et al. 2003; Loveless and Hamrick 1984). Specifically, our results are compatible with past population isolation in refugia during dry Pleistocene glacial periods and subsequent recolonisation in response to climatic oscillations (Charles‐Dominique et al. 1998; De Granville 1982). Our demographic history analysis indeed suggests genetic bottlenecks in all three genetic groups dating to the Pleistocene, but more likely to the previous‐to‐last glacial period (ca. 100,000 years ago) than to the last glacial maximum, and subsequent recovery from these bottlenecks in the east and central groups. Various areas of refuge during the Pleistocene have been suggested in French Guiana (De Granville 1982; Guitet, Pélissier, et al. 2015), which may correspond to the locations of the genetic groups identified here. In addition, environmental barriers such as rivers may have limited colonisation or homogenising gene flow (Nazareno et al. 2017), with a potential role of the Approuague river keeping the central and eastern genetic groups distinct.

### 4.2 Genomic Signatures of Selection and Their Environmental Drivers

Our analysis indicated only a moderate level of consensus among methods regarding the SNPs putatively under selection, suggesting local adaptation in *D. guianensis* is a multifarious, complex process. Partial RDA results revealed that environmental variables explained a large proportion of genetic variance among sampled sites in the French Guiana permanent forest domain (DFP), outweighing the impact of geographical factors and demographic history (genetic group membership). Similarly strong effects of climatic variables have been observed on the genetic structure of other tropical and subtropical trees, suggesting local adaptation of their populations (Flores et al. 2023; Sekely et al. 2024; Steane et al. 2014). Among the five environmental variables retained for association with genetic variation in our study, PCQ, AI, and TWI had the highest coefficients of variation among sites (17.5%, 10.9% and 7.2%), whereas among‐site variation in PETCQ and MTCQ only reached 1.5% and 1.3% (Table S1). The genetic associations with the larger environmental gradients among sites observed for PCQ, TWI, and AI suggest conditions of water stress as evolutionary drivers (excess water or drought), whereas temperature or evapotranspiration in the cooler, rainy season vary only little among sites and their roles as evolutionary drivers are more difficult to interpret biologically. Our results suggest that heavy rainfall during the rainy season, especially the pronounced rainfall gradient between east and west French Guiana, is a stronger driver of local adaptation in *D. guianensis* than drought. The physiological processes associated with selection through heavy rainfall could relate to coping with excessive moisture, for example, tolerance of hypoxia (Lopez and Kursar 2003), leaf and root trait adaptation, or effective nutrient uptake and storage mechanisms protective against soil leaching (Wang et al. 2021, Quinto‐Mosquera and Moreno‐Hurtado 2016; Wang et al. 2021; Zhang 2016). Seasonal droughts are known to trigger adaptive trait responses in tropical rainforest trees (Bonal et al. 2016). Our results suggest that at the within‐species level in *D. guianensis* , adaptive genetic response to drought may be more limited, which supports the previously described drought sensitivity of the species (Baraloto et al. 2007; Maréchaux et al. 2015; Ziegler et al. 2019). Similarly, genetic variation for drought resistance was found to be low in a set of Panamanian rainforest trees (Comita et al. 2024).

The large amount of unexplained variance in our pRDA also suggests the presence of other factors not included in the model. These may include fine local‐scale environmental variation, such as soil composition and nutrient availability, which are critical drivers of tropical tree adaptation (Brousseau et al. 2015, 2021). Topographic position, such as whether individuals are located on slopes, plateaus or in lowland areas, may also trigger adaptations by influencing microclimatic conditions and water availability. For the genera *Symphonia* and *Eschweilera*, the TWI was shown to structure functional leaf trait variation and genetic variation within and among genera (Schmitt et al. 2020, 2021). Other environmental conditions, such as neighbour competition and light availability, have been shown to structure genetic diversity of tropical trees on the microenvironmental scale (Schmitt et al. 2022). Historical demographic processes, including past population bottlenecks or founder effects, could contribute to the genetic patterns observed independently of current environmental conditions (Barthe et al. 2017; Bonnier et al. 2024; Loveless 1992). In addition, the polygenic control of many adaptive traits and genetic architectures that may be environment dependent (de Miguel et al. 2022), as suggested by gene‐pool specific selective sweeps in our study, may contribute to unexplained variance. The diversity of spatial, temporal and genetic factors driving genetic diversity in tropical trees emphasises the need for considering varied sources of information for a comprehensive design of conservation strategies.

Local adaptation to the environment is a little‐studied issue in tropical forest trees (Collevatti et al. 2019), with few studies highlighting a diversity of factors as presented in our study. Nelson et al. (2021) demonstrated that populations of *Theobroma cacao* show significant molecular adaptations in response to local pathogenic pressures. Barton et al. (2020) studied how tropical trees adapted to varying humidity conditions, revealing that populations from historically wetter sites have distinct genetic profiles from those in drier areas. It was also shown that tropical trees can adapt to seasonal variations through contrasting growth patterns influenced by these changes, indicating that long‐term environmental fluctuations play a crucial role in shaping local adaptation strategies (Enquist and Leffler 2001). These results underline the importance of understanding the diverse adaptive responses of tropical trees in order to preserve and effectively manage these critical ecosystems in the face of environmental change. It is therefore essential to further develop this type of research to better prepare us to face and reduce the impacts of climate change on tropical forests.

### 4.3 Climate Change Risk for *Dicorynia guianensis*

Our results revealed distinct spatial patterns of genomic offset and adaptive capacity across French Guiana, highlighting significant risks in western and central populations (i.e., Nouragues Inselberg) of *Dicorynia guianensis*. The main climatic factors identified in our study were changes in precipitation during the coldest quarter (PCQ) and potential evapotranspiration during the wettest quarter (PETWQ). These results highlight the role of precipitation in the local adaptation of tropical tree species, as extreme precipitation patterns and seasonal variability have profound impacts on physiological and ecological processes (Alfaro‐Sánchez et al. 2017; Bawa and Dayanandan 1998). Similar studies on maladaptation have highlighted temperature variations as the primary factor driving genomic offset (Jia et al. 2020), while also emphasising the significant roles of precipitation variability (Jordan et al. 2017; Müller et al. 2023) and photoperiod changes (Sekely et al. 2024). Western populations of *D. guianensis* , characterised by low long‐term effective population size and lower genetic diversity at putatively adaptive loci than other groups, appear to be particularly vulnerable to increased rainfall variability, which could exacerbate the risks of maladaptation and reduced fitness. Rainfall not only influences water availability, but also interactions with other environmental stressors, such as nutrient cycling, pest outbreaks and habitat connectivity (Milici et al. 2020). For *D. guianensis* , high rainfall variability likely contributes to selective pressures on traits related to water‐use efficiency and resistance to embolism, further accentuating its sensitivity to hydrological changes (Ziegler et al. 2019).

The use of two distinct socio‐economic pathways (SSP2‐4.5 and SSP3‐7.0) in our genomic offset models provided consistent predictions, with both scenarios producing similar patterns of maladaptation risk. This demonstrates that, under varying future climatic scenarios, the populations at risk of maladaptation remain largely the same. Such insights highlight that, regardless of the specific trajectory of climate change within plausible ranges, certain populations are likely to face maladaptation risks. This robustness in identifying vulnerable populations underscores the utility of genomic offset analyses for prioritising conservation and management actions in areas with heightened vulnerability (Capblancq et al. 2018, 2020). However, limitations remain, including the potential oversimplification of adaptive traits and environmental interactions, as well as uncertainties associated with future climate variability and local environmental heterogeneity (Lind et al. 2024; Rellstab et al. 2021). Genomic offset models also rely heavily on the resolution and accuracy of climatic data, as well as the environmental variables selected for analysis. Variation in these factors can influence the predicted magnitude of maladaptation and the specific populations identified as most at risk (Rellstab et al. 2015). To address these challenges, integrating genomic offset analyses with experimental studies, such as reciprocal transplant experiments or common garden trials, would provide valuable evaluations of the predictive accuracy of genomic offset models through their relationship to fitness traits under varying climatic conditions (Fitzpatrick et al. 2021). This combined approach would offer deeper insights into the resilience and conservation needs of tropical tree species like *D. guianensis* , ensuring sustainable management under the dual pressures of climate change and anthropogenic exploitation.

### 4.4 Conservation Recommendations

To ensure the sustainable management of *Dicorynia guianensis*, it is essential to integrate adaptive strategies that balance ecological and economic goals. The current PRFB guidelines (PRFB 2019), such as limiting harvest to five trees per hectare and implementing a 65‐year rotation, should be revised based on genomic data by using region‐specific guidelines to include adaptive logging quotas. These quotas should be lower in areas identified as having high genomic offset or low adaptive capacity, such as western French Guiana. Additionally, certain geographical groups with significant vulnerability should be designated as genomic protection zones, where logging is either strictly regulated or prohibited altogether. According to our results, locations like Apatou and its surrounding forested areas can be candidates for genomic protection. The establishment of mandatory genetic assessments before granting timber harvesting permits in at‐risk regions would further sustainable practices (Allendorf et al. 2022). Quantifying the within and among provenance components of survival and growth in reciprocal transplantation trials could inform on the usefulness of implementing an assisted gene flow project to supplement areas at higher risk of climate‐induced maladaptation with seeds from geographical groups not at risk. This would offer a proactive solution to enhancing genetic diversity in geographical groups at risk, particularly under shifting climatic conditions (Weeks et al. 2011). Seed banks should also be developed to preserve genetic diversity (FAO 2020) and support restoration initiatives in degraded forest areas, for example following gold mining operations (Grimaldi et al. 2015). Another key measure is the diversification of timber species to reduce the logging pressure on *D. guianensis* . Timber markets should be encouraged to adopt and promote alternative species by establishing quality and market value certification frameworks (Schulze et al. 2022). Education campaigns targeting timber users and suppliers can further encourage the use of diverse species. In addition, support for research into the commercial viability and ecological roles of lesser‐used species will help to ensure the long‐term sustainability of forests (Heuertz et al. 2023; Holderegger et al. 2019). Furthermore, the interaction between selective logging and the sensitivity of *D. guianensis* to drought warrants further study to understand how drought stress and canopy disturbance may exacerbate each other (Fargeon et al. 2016; Hérault and Gourlet‐Fleury 2016).

## Author Contributions

Designed research: J.B., O.B., S.T., N.T., M.H.; performed research: J.B., E.S.L., T.F., V.T., S.S., N.T., M.H.; contributed new reagents or analytical tools: E.S.L., T.F., S.S.; analysed data: J.B., E.S.L., T.F.; wrote the paper: J.B., N.T., M.H. All authors critically read and approved the submitted version of the paper.

## Disclosure

Benefit‐Sharing Section: This research complies with the national laws of French Guiana and France implementing the Convention on Biological Diversity and the Nagoya Protocol. Benefits generated include: Sharing of research and development results through open access publication, and FAIR principles for data, collaboration, cooperation and contribution in scientific research in the Party providing genetic resources, that is, scientists from French Guiana are included as co‐authors on this study. The sharing of research findings with local stakeholders, including the ONF (Office National des Forêts), to support sustainable forest management initiatives. Contributions to a broader understanding of *Dicorynia guianensis* genetics and ecology, aiding its conservation and sustainable use.

## Supporting information


Data S1.

## Data Availability

The study project data are registered in the European Nucleotide Archive (ENA) under the project accession number: PRJEB83334. *Genetic data*: Fastq files generated for this study, are deposited in the European Nucleotide Archive (ENA) under accession numbers ERA31116056 (fastq). *Sample metadata*: Metadata, including georeferences in decimal degrees and sampling dates, is stored on the ENA (accession number ERA31035407).
