# Supplementary material for: Wet Season Environments Drive Local Adaptation in the Timber Tree Dicorynia guianensis in French Guiana
Source: Mol Ecol. 2025 Apr 8;34(23):e17759. doi: 10.1111/mec.17759 (PMC12684327; doi:10.1111/mec.17759)
Supplement: Supplementary file 1 — Data S1. [file MEC-34-e17759-s001.docx]

**Supporting Information for:**

**Wet season environments drive local adaptation in the timber tree *Dicorynia guianensis* in French Guiana**

Julien Bonnier^1,2^, Enrique Sáez Laguna^1^, Thomas Francisco^1^, Valérie Troispoux^2^, Olivier Brunaux^3^, Sylvain Schmitt^4^ Stéphane Traissac^2^, Niklas Tysklind^2^ & Myriam Heuertz^1^

^1^BIOGECO, INRAE, Univ. Bordeaux, 69 route d’Arcachon, 33610 Cestas, France

^2^ECOFOG, INRAE, Agroparistech, CNRS, Cirad, Université des Antilles, Univ. de la Guyane, 97310 Kourou, French Guiana

^3^ONF, R&D, Réserve de Montabo, BP 7002, 97307 Cayenne Cedex, French Guiana

^4^UPR Forests and Societies, CIRAD, Montpellier France

Julien Bonnier: <https://orcid.org/0000-0003-2009-6743>, [julien.bonnier@inrae.fr](mailto:julien.bonnier@inrae.fr)

Enrique Sáez Laguna: <https://orcid.org/0009-0003-3836-9962>, [enrique.saez-laguna@u-bordeaux.fr](mailto:enrique.saez-laguna@u-bordeaux.fr);

Thomas Francisco: <https://orcid.org/0009-0005-8618-0747>, [thomas.francisco@inrae.fr](mailto:thomas.francisco@inrae.fr)

Valérie Troispoux: <https://orcid.org/0000-0002-1118-1367>, valerie.Troispoux@ecofog.gf

Olivier Brunaux: <https://orcid.org/0000-0002-3392-0423>, [olivier.brunaux@onf.fr](mailto:olivier.brunaux@onf.fr)

Sylvain Schmitt: <https://orcid.org/0000-0001-7759-7106>; [sylvain.m.schmitt@gmail.com](mailto:sylvain.m.schmitt@gmail.com)

Stéphane Traissac: <https://orcid.org/0000-0001-9255-1616>, [stephane.traissac@agroparistech.fr](mailto:stephane.traissac@agroparistech.fr)

Niklas Tysklind: <https://orcid.org/0000-0002-6617-7875>, [niklas.tysklind@inrae.fr](mailto:niklas.tysklind@inrae.fr)

Myriam Heuertz <https://orcid.org/0000-0002-6322-3645>, [myriam.heuertz@inrae.fr](mailto:myriam.heuertz@inrae.fr)

| **Groups** | **Sites names** | **lat** | **long** | **Elevation (m)** | **PET Wettest Quarter** | **Aridity Index** | **Topographic Wetness Index** | **Precipitation Coldest Quarter (mm)** | **Mean Temperature Coldest Quarter (°C)** |
| --- | --- | --- | --- | --- | --- | --- | --- | --- | --- |
| **West** | **Acarouany** | 5.5441 | -53.8123 | 49 | 123.21 | 39.43 | 12.05 | 644 | 25.6 |
|  | **Apatou** | 5.2384 | -54.2665 | 35 | 123.84 | 30.93 | 11.96 | 619 | 25.7 |
| **East** | **MC_88** | 4.0544 | -52.0723 | 95 | 120.97 | 39.01 | 12.09 | 995 | 25.1 |
|  | **MC_87** | 4.0571 | -52.0861 | 108 | 120.72 | 38.13 | 12.18 | 1074 | 25.1 |
|  | **Foret Regina St Georges** | 4.0840 | -52.1645 | 35 | 120.43 | 33.34 | 12.22 | 1014 | 25.1 |
|  | **Saut Lavillette** | 4.1579 | -52.2062 | 32 | 120.46 | 43.18 | 11.58 | 1087 | 25.2 |
| **Central** | **Cacao** | 4.5497 | -52.5003 | 4 | 118.25 | 31.91 | 11.11 | 956 | 25.3 |
|  | **Nouragues Inselberg** | 4.1033 | -52.6832 | 95 | 118.57 | 31.67 | 9.89 | 971 | 24.4 |
|  | **Piste St Elie** | 5.3349 | -53.0372 | 43 | 119.88 | 37.62 | 12.45 | 857 | 25.1 |
|  | **Regina** | 4.3706 | -52.3187 | 83 | 118.47 | 33.63 | 10.51 | 961 | 25.0 |
| **St Georges** | **St Georges** | 4.0021 | -51.9583 | 73 | 121.01 | 35.19 | 12.52 | 1086 | 25.1 |
|  | **Coefficient of variation of env. variables (Std/mean, in %)** |  |  |  | 1.5 | 10.9 | 7.2 | 17.5 | 1.3 |

**Table S1.** Table of environmental variable values, location and elevation for studied sites of *Dicorynia guianensis* in French Guiana.

|  | **Acarouany** | **Apatou** | **Cacao** | **Foret Regina St Georges** | **MC_87** | **MC_88** | **Nouragues Inselberg** | **Piste St Elie** | **Regina** | **Saut Lavillette** |
| --- | --- | --- | --- | --- | --- | --- | --- | --- | --- | --- |
| **Apatou** | 0.015** |  |  |  |  |  |  |  |  |  |
| **Cacao** | 0.129*** | 0.136*** |  |  |  |  |  |  |  |  |
| **Foret Regina St Georges** | 0.097*** | 0.102*** | 0.063 |  |  |  |  |  |  |  |
| **MC_87** | 0.113*** | 0.121*** | 0.085** | 0.065** |  |  |  |  |  |  |
| **MC_88** | 0.09*** | 0.096*** | 0.036** | 0.023** | 0.051* |  |  |  |  |  |
| **Nouragues Inselberg** | 0.136*** | 0.142*** | 0.022** | 0.061** | 0.089** | 0.04** |  |  |  |  |
| **Piste St Elie** | 0.139*** | 0.146*** | 0.025** | 0.071*** | 0.092 | 0.042** | 0.023** |  |  |  |
| **Regina** | 0.123*** | 0.129*** | 0.008 | 0.046** | 0.079** | 0.021** | 0.02** | 0.017** |  |  |
| **Saut Lavillette** | 0.084*** | 0.088*** | 0.071*** | 0.031** | 0.060 | 0.025** | 0.072 | 0.079*** | 0.059** |  |
| **St Georges** | 0.080*** | 0.085*** | 0.026** | 0.032** | 0.050 | 0.015** | 0.039** | 0.042** | 0.025** | 0.032** |

**Table S2.** *F*_ST_ distances between *Dicorynia guianensis* sampling sites, * p < 0.05; p < 0.01; p < 0.001, the significance of the values was obtained by performing 999 bootstrap replicates.

| **Sampling sites** | ***N*** | ***H*_O_** | ***H*_E_** | ***F*_IS_** | ***A*_R_** | ***π*** |
| --- | --- | --- | --- | --- | --- | --- |
| **Acarouany** | 8 | 0.128 | 0.203 | 0.281 | 1.51 | 0.099 |
| **Apatou** | 8 | 0.114 | 0.164 | 0.262* | 1.41 | 0.041 |
| **Cacao** | 6 | 0.228 | 0.290 | 0.171* | 1.69 | 0.134 |
| **Foret_Regina_St_Georges** | 7 | 0.261 | 0.359 | 0.250* | 1.80 | 0.170 |
| **MC_87** | 4 | 0.253 | 0.381 | 0.270* | 1.80 | 0.244 |
| **MC_88** | 8 | 0.240 | 0.388 | 0.363 | 1.83 | 0.215 |
| **Nouragues_Inselberg** | 15 | 0.174 | 0.291 | 0.382 | 1.71 | 0.168 |
| **Piste_St_Elie** | 7 | 0.211 | 0.356 | 0.352 | 1.78 | 0.253 |
| **Regina** | 8 | 0.209 | 0.312 | 0.290* | 1.72 | 0.153 |
| **Saut_Lavilette** | 8 | 0.251 | 0.348 | 0.238* | 1.78 | 0.170 |
| **St_georges** | 8 | 0.285 | 0.406 | 0.269* | 1.87 | 0.231 |
| **East** | 27 | 0.250 | 0.384 | 0.353* | 1.92 | 0.126 |
| **Central** | 36 | 0.198 | 0.329 | 0.395 | 1.89 | 0.128 |
| **West** | 16 | 0.121 | 0.190 | 0.294 | 1.62 | 0.073 |

**Table S3.** **Genetic diversity statistics for sampling sites and groups of *Dicorynia guianensis* based on 459 loci putatively under selection identified by at least four methods.** N, sample size; *A*_R_, allelic richness standardized to a sample size of 12 gene copies; *H*_E_, expected heterozygosity; *H*_O_, observed heterozygosity; *F*_IS_, inbreeding coefficient; π, nucleotide diversity per SNP across 459 SNP loci putatively under selection (multiplied by 1000); *, P < 0.05; **, P < 0.01; ***, P < 0.001.


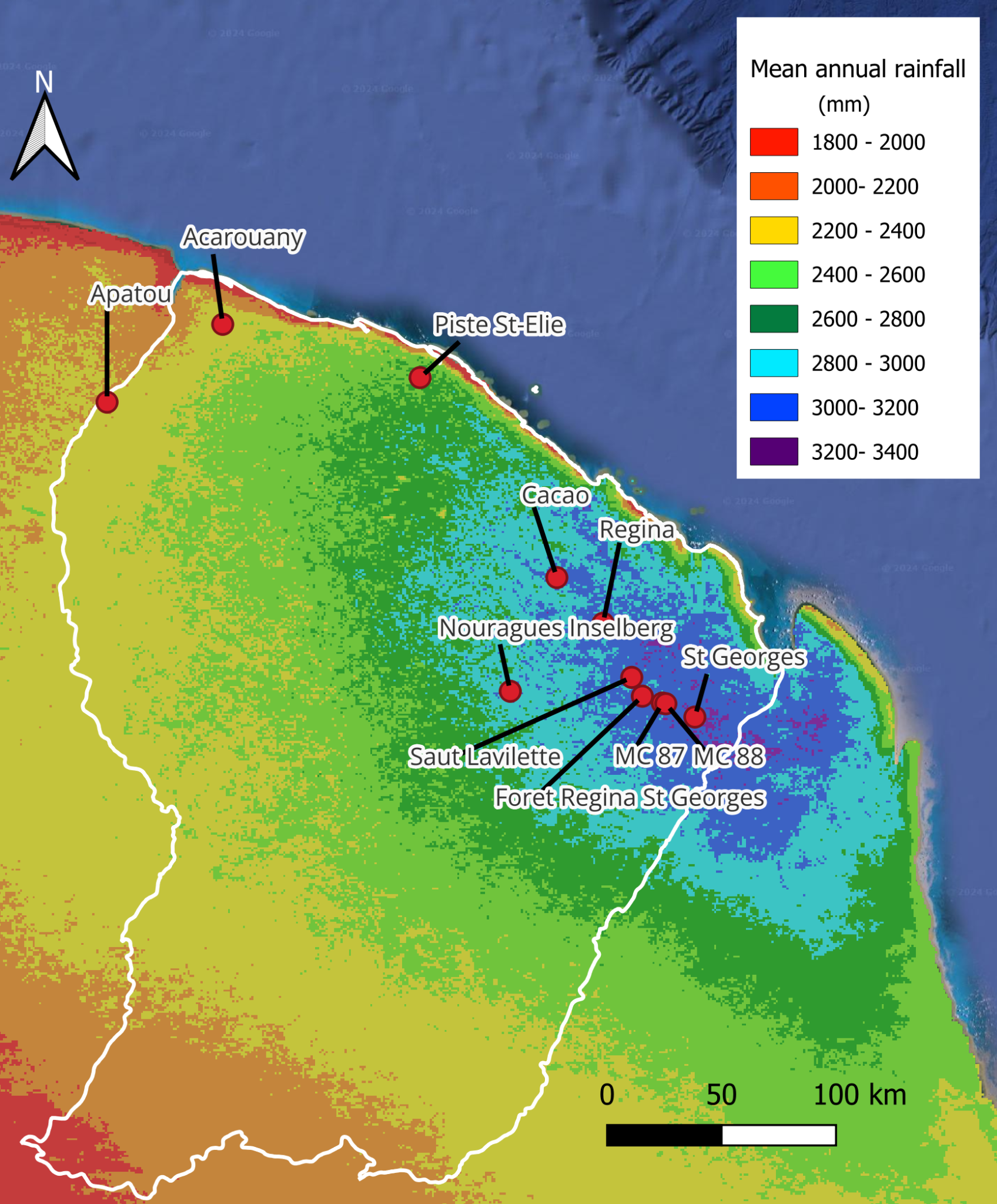


**Figure S1.** Map of mean annual rainfall in French Guiana. This map is based on WorldClim bioclimatic variables from 1970 to 2000.


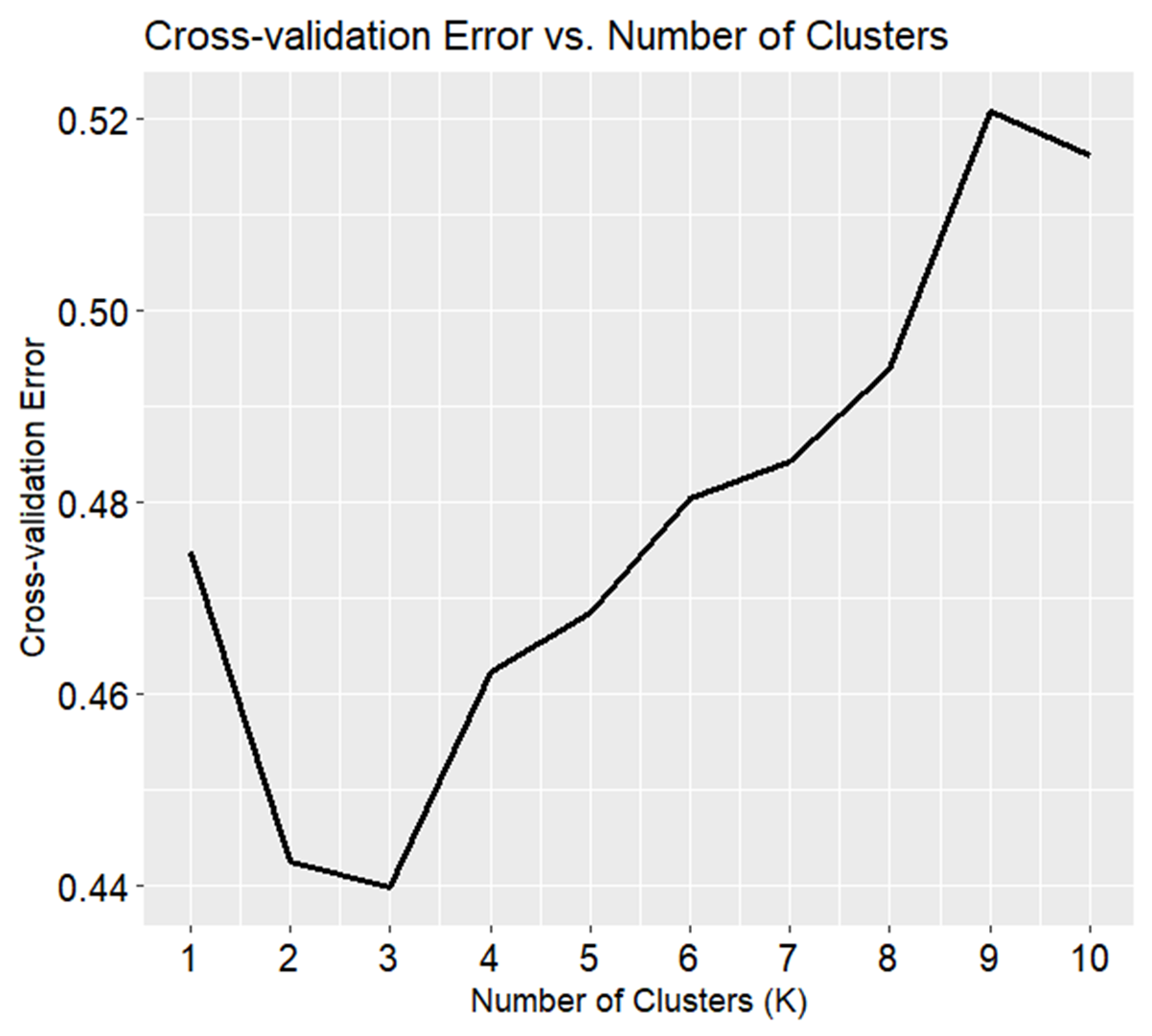


**Figure S2.** Plot of ADMIXTURE cross validation error from K=1 through K=10. We choose K=3, with the minimal value of cross validation error.

**
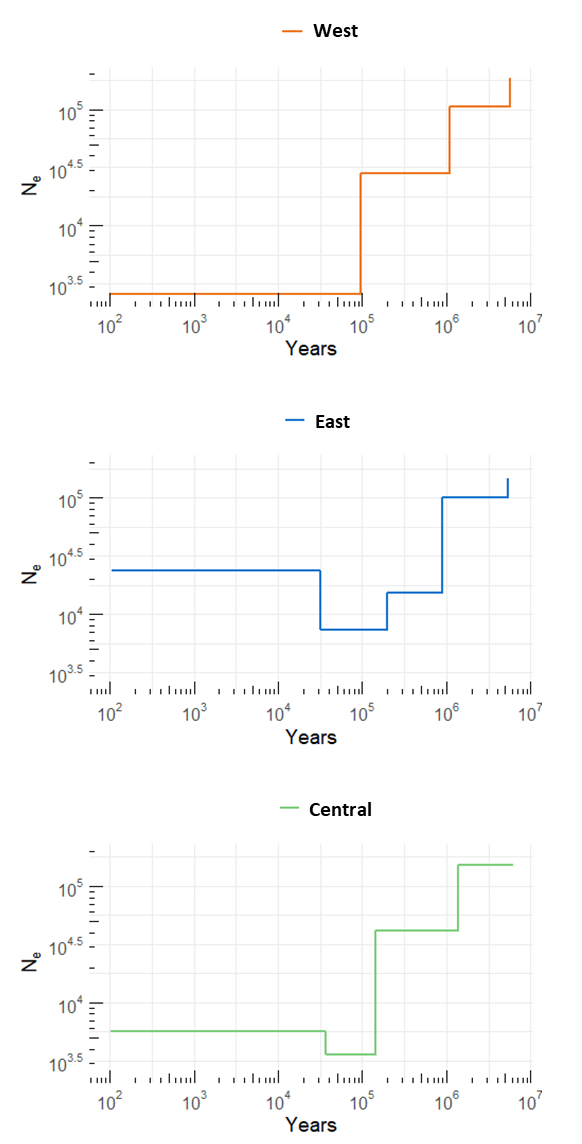
**

**Figure S3.** Stairway plot showing changes in effective population size (*N_e_*) through years of three groups of sampling sites (West, East, Central).

**
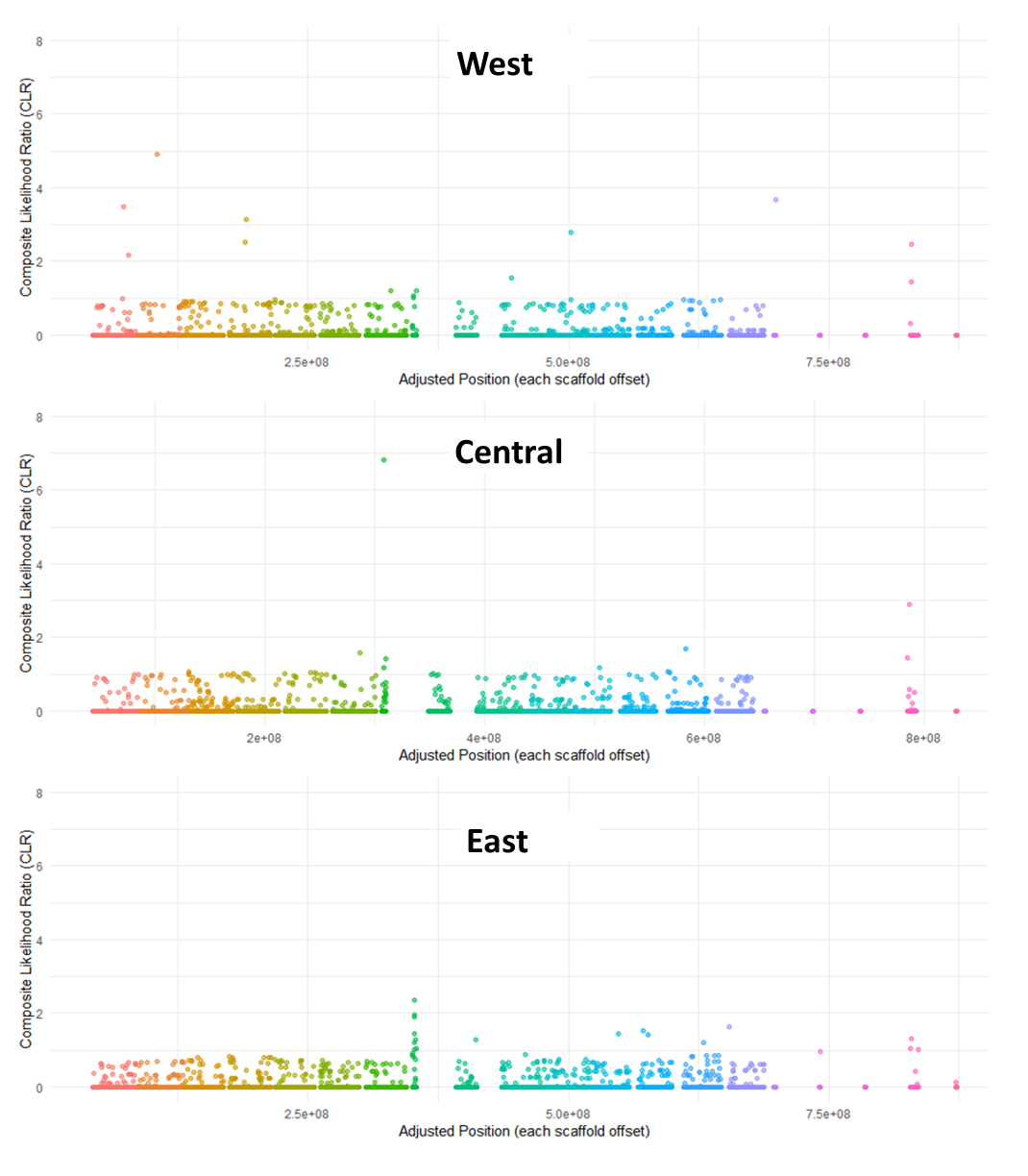
**

**Figure S4.** Scan of the *D. guianensis* genome for selective sweeps using SweeD. Manhattan plots showing SNPs with CLR >0 on the 20 largest *Dicorynia guianensi*s super-scaffolds for the three groups. Each color represents a different super- scaffold.

**
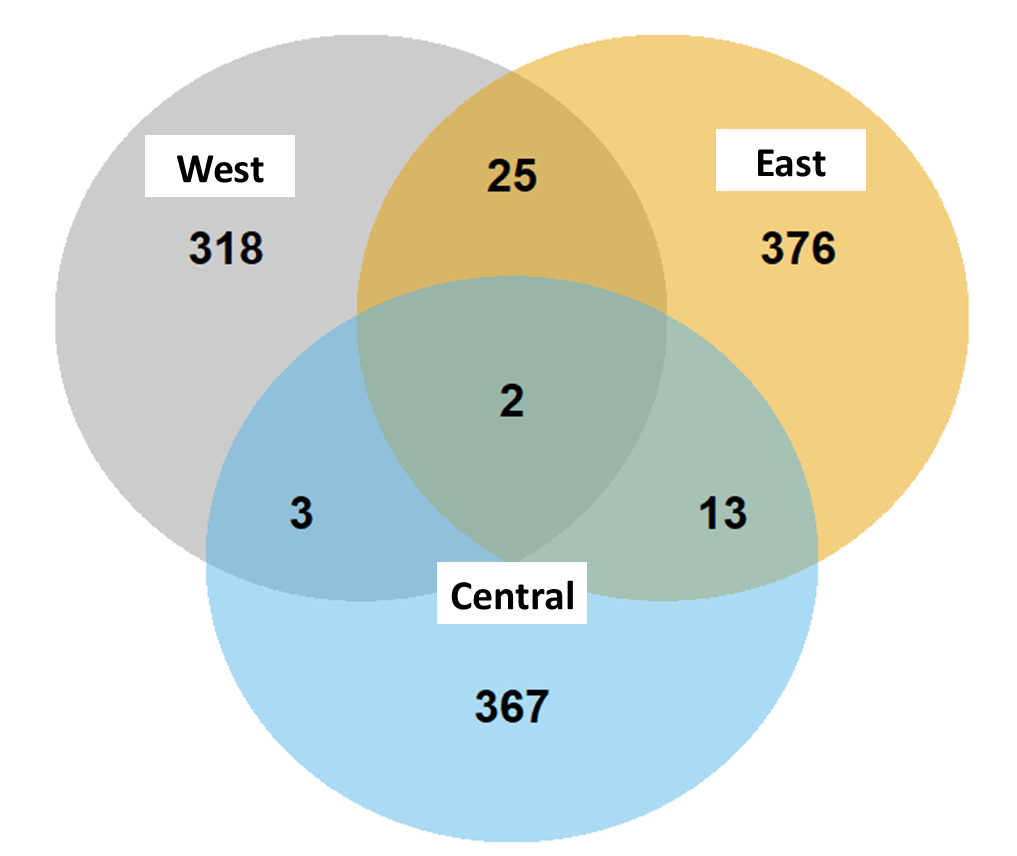
**

**Figure S5.** Venn diagram showing SNPs with CLR >0 for SNPs affected by selective sweeps using SweeD analysis on the 20 largest *Dicorynia guianensi*s super-scaffolds for the three groups.


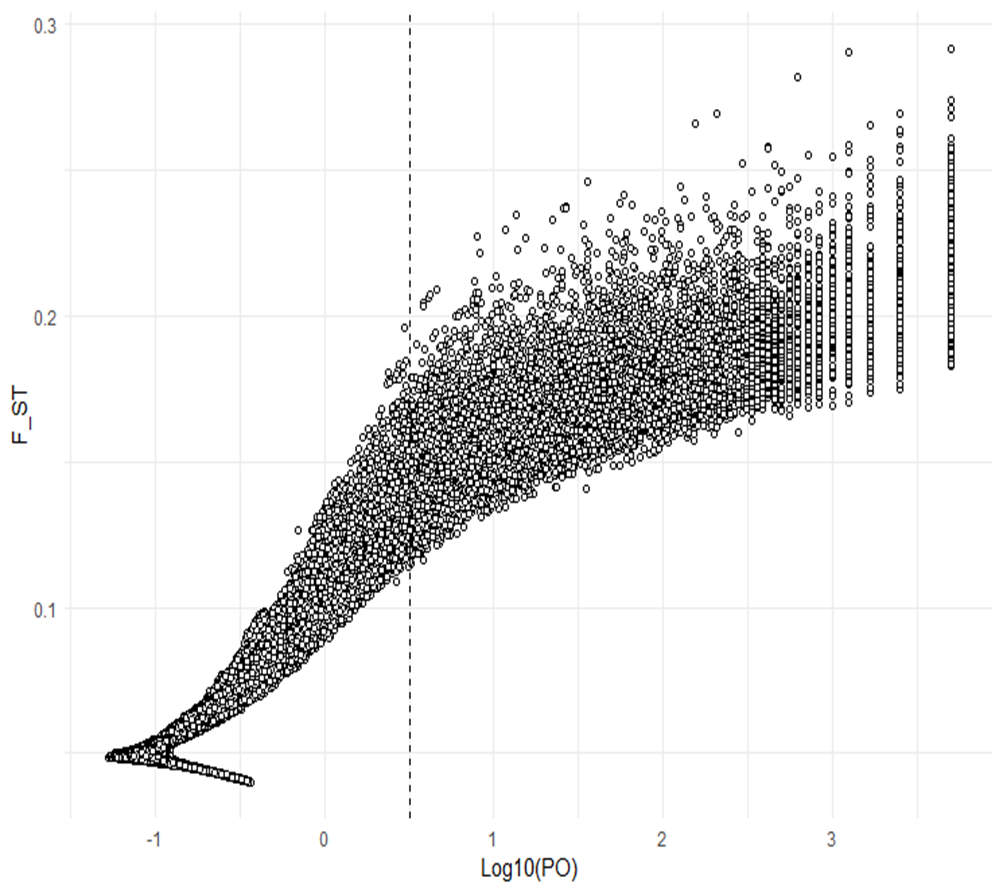


**Figure S6**. Plot of FST values and Bayes factors (log10) obtained using the BayeScan outlier test. Dashed lines indicate the Bayes factor threshold of 0.5 (log10).

**
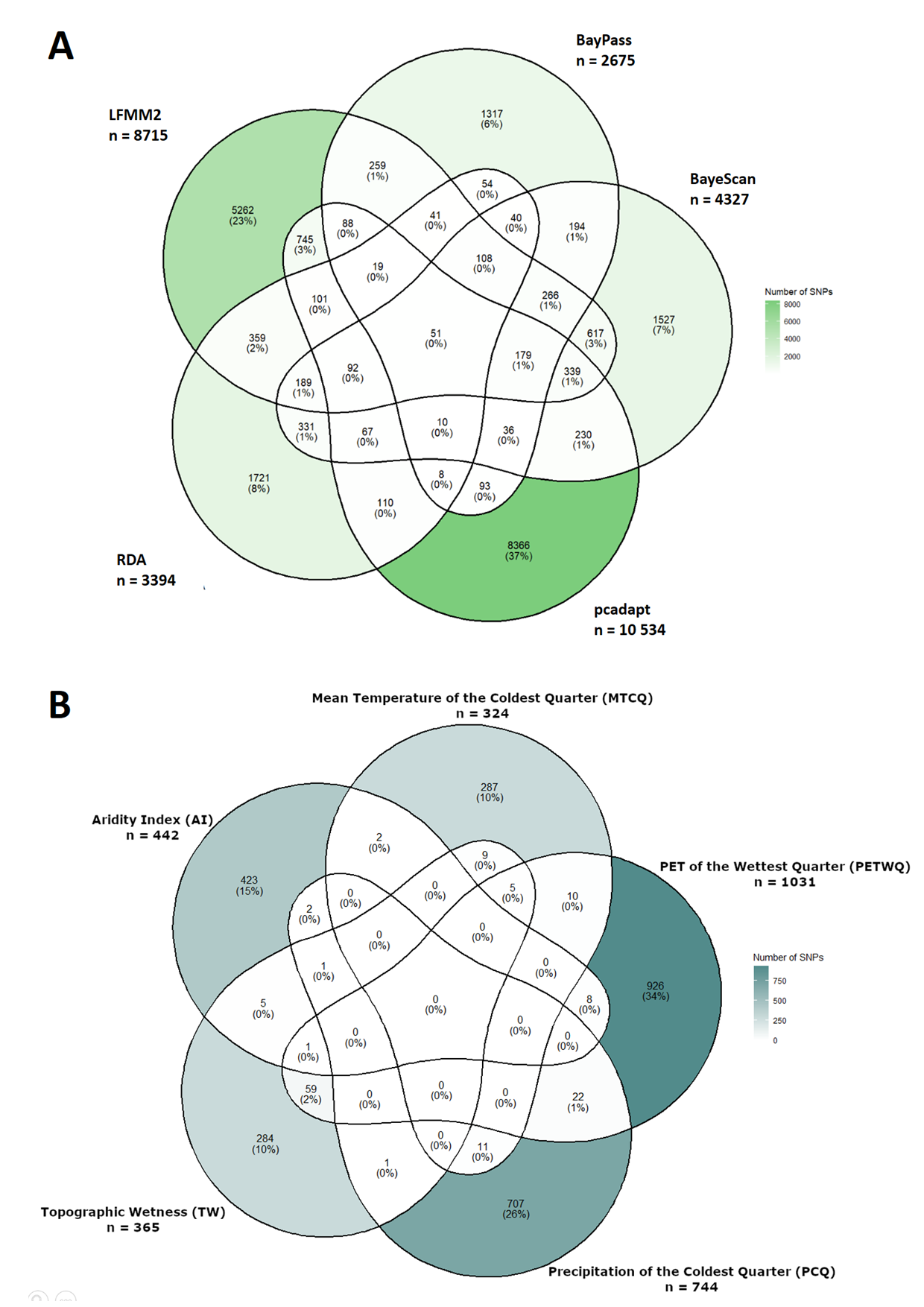
**

**Figure S7.** Overlap and unique contributions of genome analysis methods and environmental associations in *Dicorynia guianensis*. Overlapping sections of the Venn diagrams represent SNPs flagged as putatively under environmental selection according to a shared set of methods or variables, while non-overlapping sections represent SNPs unique to specific methods or variables. Percentages indicate the proportion of SNPs relative to the total number analyzed. **A.** Overlap between significant SNPs identified by each genome analysis method. **B.** Number of SNPs significantly associated with each of the five environmental variables in the BayPass gene-environment association test. Each environmental variable is labeled outside the Venn diagram, with the number of SNPs associated with it.

**Figure S8.** Bubble plots of GO term enrichment analysis for *Dicorynia guianensis* genes identified by outlier analyses in biological processes, cellular components and molecular functions. Plots show the 15 most significant represented terms per category.
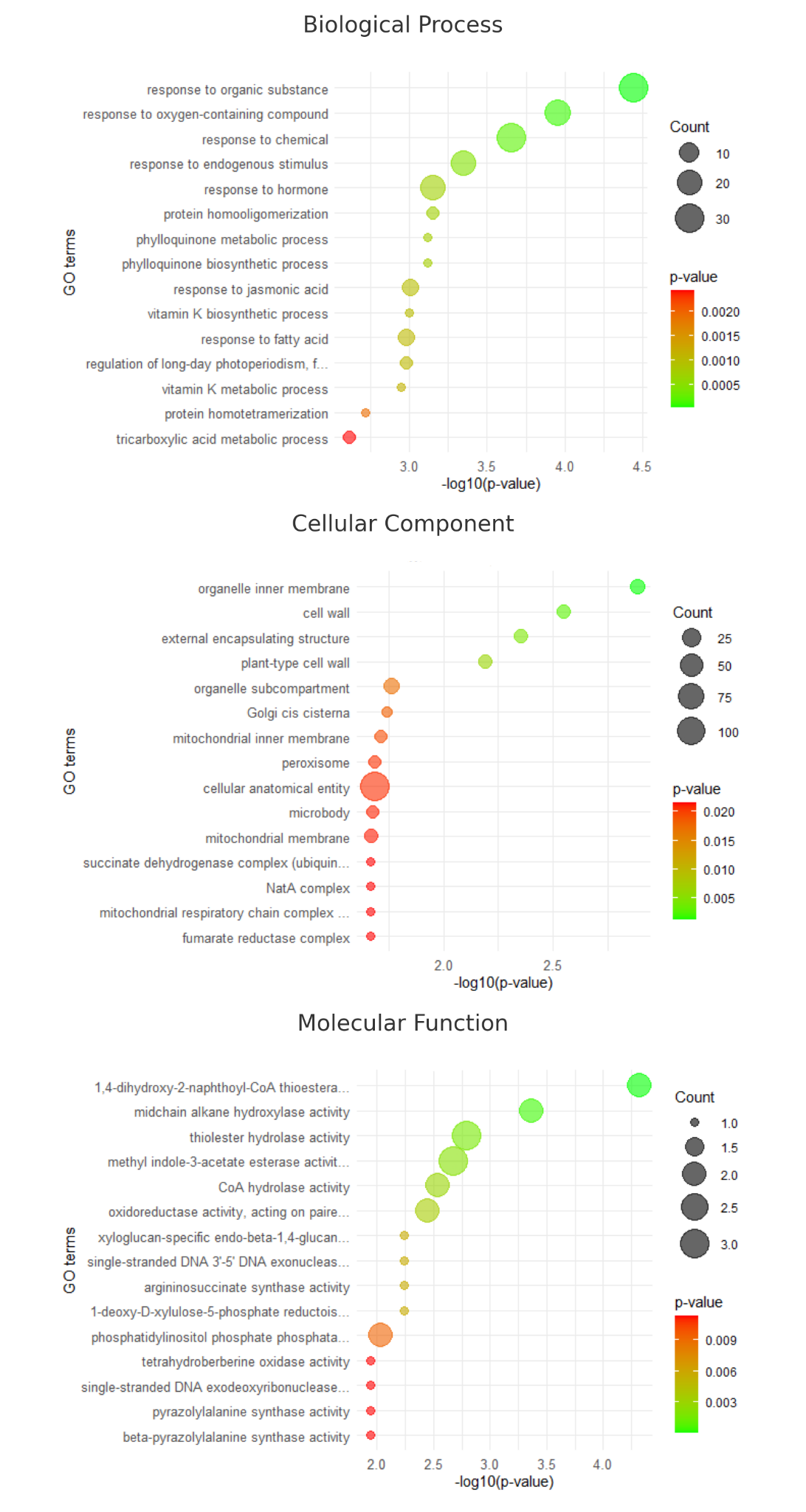


**Figure S9.** Climate model projection used in genomic offset analysis of *Dicorynia guianensis* for three different study areas: French Guiana, the Domaine Forestier Permanent (DFP) and a restricted area of the DFP based on sampling sites interpolation.
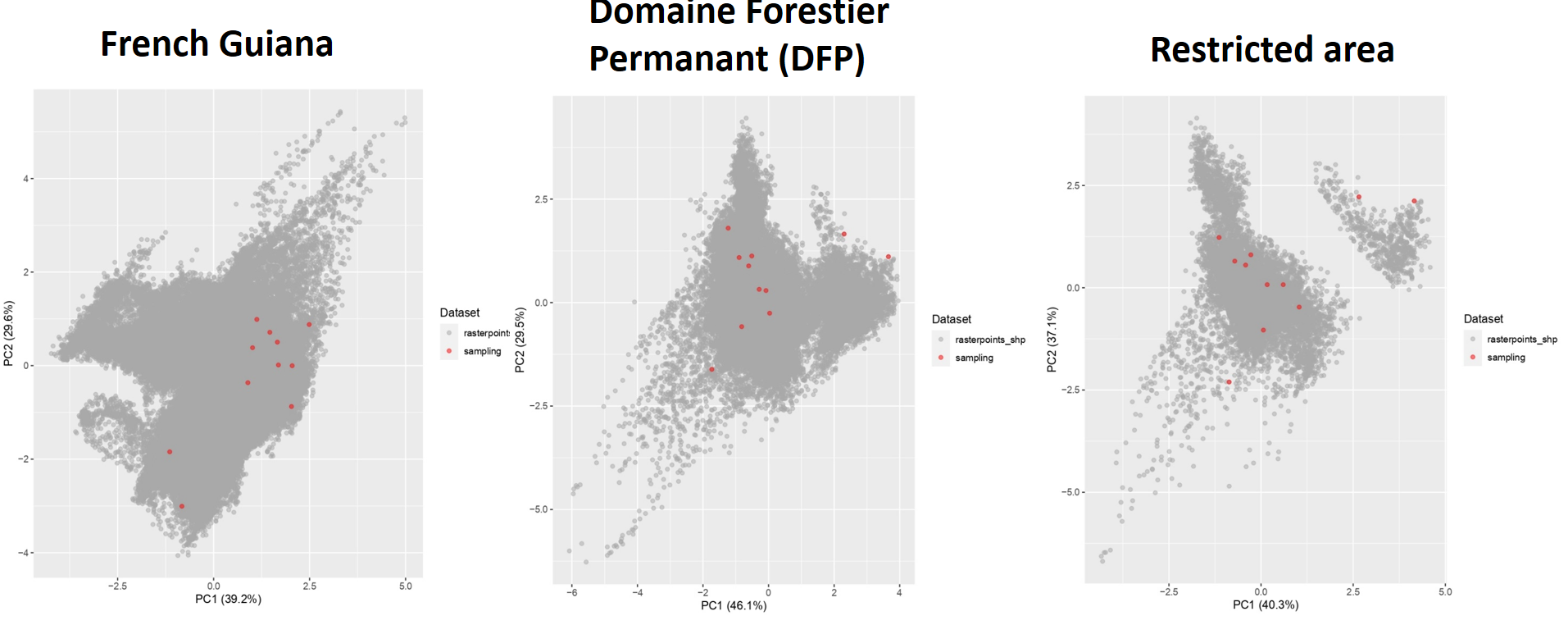


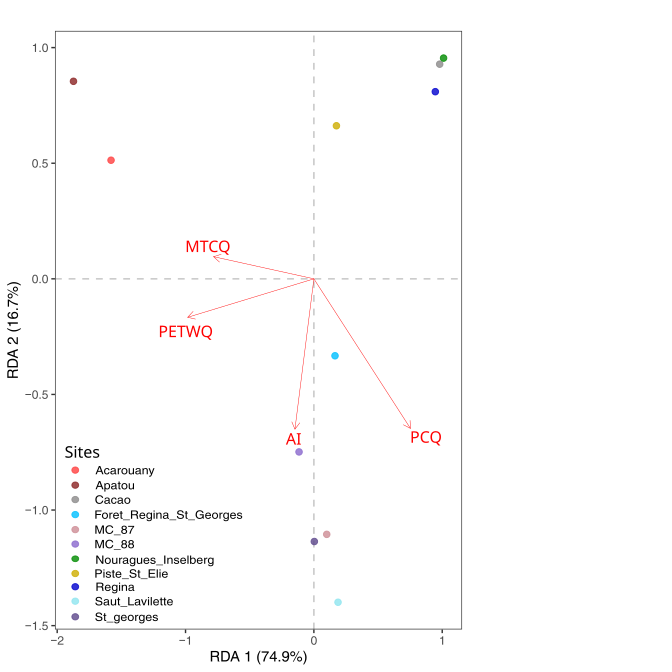


**Figure S10**. Biplot of redundancy analysis (RDA) illustrating the strength of association between environmental variables and genetic variation at 459 putatively adaptive loci at 11 study sites for *Dicorynia guianensis* in French Guiana. The figure highlights the relationship of sampling sites based on significant loci and specific environmental variables, emphasizing the climatic factors most strongly associated with genetic variation. Variable names: MTCQ, Mean Temperature of the Coldest Quarter; PETWQ, Potential Evapotranspiration of the Wettest Quarter; AI, Aridity Index; PCQ, Precipitation of the Coldest Quarter.


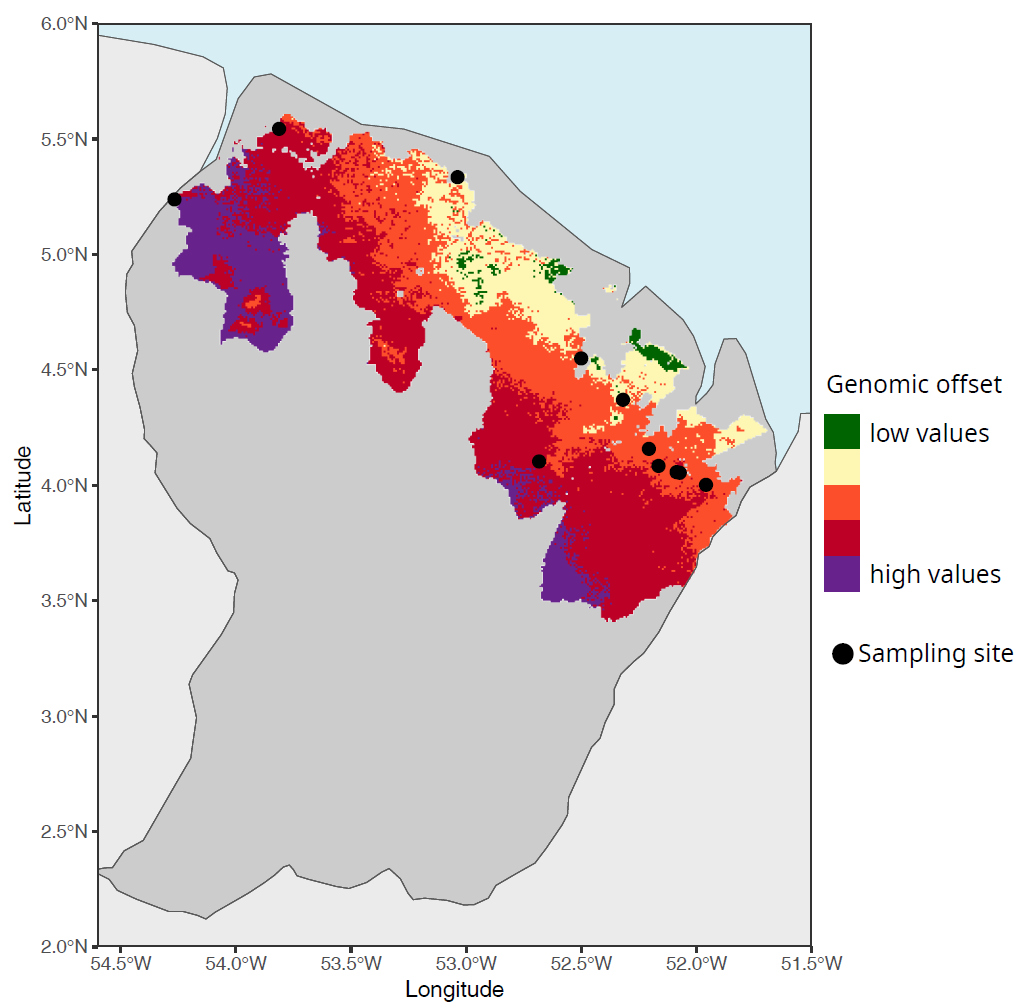


**Figure S11**. Spatial projection of the genomic offset across the Permanent Forest Domain (DFP) in French Guiana for the 2041–2060 period under the Shared Socio-economic Pathway (SSP) 3-7.0, a severe emissions scenario.
